# Zinc *K*-edge XANES spectroscopy of mineral and organic standards

**DOI:** 10.1107/S160057751900540X

**Published:** 2019-06-21

**Authors:** Erin K. Griffith, Ellery D. Ingall, Peter L. Morton, David A. Tavakoli, Barry Lai

**Affiliations:** a School of Earth and Atmospheric Sciences, Georgia Institute of Technology, 311 Ferst Dr NW, Atlanta, GA 30332-0340, USA; b Geochemistry, National High Magnetic Field Laboratory, 1800 E Paul Dirac Dr, Tallahassee, FL 32310, USA; c Materials Characterization Facility, Georgia Institute of Technology, 345 Ferst Dr NW, Atlanta, GA 30332-1000, USA; d Advanced Photon Source, Argonne National Laboratory, 9700 South Cass Avenue, Argonne, IL 60439, USA; University of Essex, UK

**Keywords:** zinc standards, Zn *K*-edge, XANES, spectroscopy

## Abstract

Zinc *K*-edge X-ray absorption near-edge (XANES) spectroscopy was conducted on 40 zinc mineral samples and organic compounds. The *K*-edge position varied from 9660.5 to 9666.0 eV and a variety of distinctive peaks at higher post-edge energies were exhibited by the materials. Zinc is in the +2 oxidation state in all analyzed materials, thus the variations in edge position and post-edge features reflect changes in zinc coordination. For some minerals, multiple specimens from different localities as well as pure forms from chemical supply companies were examined. These specimens had nearly identical *K*-edge and post-edge peak positions with only minor variation in the intensity of the post-edge peaks. This suggests that typical compositional variations in natural materials do not strongly affect spectral characteristics. Organic zinc compounds also exhibited a range of edge positions and post-edge features; however, organic compounds with similar zinc coordination structures had nearly identical spectra. Zinc XANES spectral patterns will allow identification of unknown zinc-containing minerals and organic phases in future studies.

## Abstract 2

Zinc *K*-edge XANES reference standards of zinc mineral samples and organic compounds are presented.

## 1. Introduction

Zinc *K*-edge X-ray absorption near-edge spectroscopy (XANES) is a valuable tool for determining the solid-phase chemical speciation of zinc across a wide range of disciplines and applications. Agriculturally, research includes using zinc to enhance the grain concentration of plants (Doolette *et al.*, 2018 ▸) and using colloidal particles to increase the rate of discharge of zinc and phospho­rus from swine manure used as a fertilizer (Yamamoto & Hashimoto, 2017 ▸). Industrial studies have looked at the speciation of zinc in a fly ash spill (Rivera *et al.*, 2017 ▸) and steelmaking sludge (Wang *et al.*, 2013 ▸). Environmentally, there has been research into heavy metals in combined sewer overflow discharge (Rouff *et al.*, 2013 ▸) and organic waste amended soil as fertilizer (Tella *et al.*, 2016 ▸; Mamindy-Pajany *et al.*, 2014 ▸). Because XANES measures structural composition, other research has included multiple nanotechnology studies (Zhou *et al.*, 2017 ▸; Frenkel *et al.*, 2001 ▸). However, research is not limited to environmental and industrial applications, as zinc *K*-edge XANES has also been used to analyze the paint in historic paintings, without degrading the pigments (Gervais *et al.*, 2015 ▸).

Zinc, as a bioactive trace metal, is a vital nutrient for all living organisms, yet can also be toxic at high concentrations. The chemical form of zinc can be a key factor in determining its potential bioavailability or toxicity to organisms. In turn, zinc bioavailability has implications for the health and productivity of riverine (Le Pape *et al.*, 2014 ▸), terrestrial (Isaure *et al.*, 2005 ▸; Webb & Gaillard, 2015 ▸; Manceau *et al.*, 2002 ▸; Sarret *et al.*, 2004 ▸) and marine systems (Löscher, 1999 ▸). In marine environments, zinc is a component of two enzymes – carbonic anhydrase and alkaline phosphatase (Sunda & Huntsman, 2005 ▸). These enzymes are critical for the growth of most planktonic organisms serving as primary producers at the base of the food chain. In marine diatoms, which account for 20% of total global primary productivity (Malviya *et al.*, 2016 ▸), zinc is vital in the growth of their hard silica exoskeleton (Ellwood & Hunter, 2000 ▸).

A common approach in the study of elemental cycling within natural systems is to fit XANES spectra of unknown samples with spectra of known, well characterized materials (Leinweber *et al.*, 2007 ▸; Gaur *et al.*, 2009 ▸; Gräfe *et al.*, 2014 ▸; Da Silva-Cadoux *et al.*, 2012 ▸; Oakes *et al.*, 2012 ▸; Longo *et al.*, 2014 ▸). For many elemental systems, including zinc, comprehensive databases of XANES spectra are not available, which makes it more difficult to interpret spectra of unknown materials. Although plots of Zn-XANES spectra for standards of a limited number of materials may appear in studies, such as the ones listed above, the data for these spectra is typically not provided. Some previous zinc spectroscopy studies focused on standards created for EXAFS at a temperature of 20 K (Jacquat *et al.*, 2009 ▸), but a large, comprehensive set of zinc *K*-edge XANES, which would be especially relevant for measurements under environmental conditions (Monsant *et al.*, 2011 ▸), does not appear to currently exist.

The breadth of zinc standards that can be analyzed concurrently with the research being conducted is often limited by scheduling constraints. As a result, many studies must rely on a small sample set of zinc standards which is then used to inform the XANES analysis of the focus of the research (Cheng *et al.*, 2018 ▸; Wang *et al.*, 2007 ▸; Dessombz *et al.*, 2013 ▸; Adediran *et al.*, 2015 ▸). The zinc standard references presented here (Table S1 of the supporting information) will provide researchers with a diverse spectra database to initiate studies of zinc mineral speciation, thereby saving valuable synchrotron time for sample analyses. These references include both natural and synthetic materials, which will be useful in characterizing the mineral speciation of zinc in natural and anthropogenically impacted settings.

## 2. Methods

Zinc spectroscopy was run on 34 different mineral or inorganic phases and six zinc-containing organic phases. Multiple samples of some of the naturally occurring minerals were measured to evaluate the effects of compositional variations within one mineral species. The samples were either in private collections or were obtained from mineral dealers or chemical supply companies. The identity of all minerals, with the exception of one hetaerolite sample with insufficient sample mass, was verified with powder X-ray diffraction (XRD) at the Georgia Institute of Technology.

The zinc materials were measured at the 2-ID-D beamline at the Advanced Photon Source synchrotron at Argonne National Laboratories in Illinois, USA. This beamline has an energy range of 5–30 keV and a focused flux of 4 × 10^9^ photons s^−1^ at 10 keV which makes it ideal for high-resolution X-ray imaging (Ingall *et al.*, 2018 ▸). The source of the beamline is a 3.3 cm-period undulator, and a Kohzu Si (111) monochromator was used to provide an energy resolution of 1.4 × 10^−4^Δ*E*/*E*. The energy was calibrated at 9659 eV using the inflection point of the *K*-edge of a zinc metal foil standard material. The samples were ground to a fine powder (<10 µm) to minimize self-absorption. Comparing the fluorescence with the transmission data confirms that the samples were sufficiently thin to avoid self-absorption in accordance with previously accepted methods (Waychunas *et al.*, 2003 ▸). Less than 1 mg of powder was distributed over approximately 1 cm^2^ of cellulose acetate membrane which was then placed over a slot on an aluminium mounting stick. For these experiments, the undulator was tuned to 9.68 keV and the energy scan was conducted in 0.5 eV steps between 9.64 and 9.72 keV while the X-ray fluorescence signal was collected by a silicon drift detector (Vortex-EM).

Using the fluorescence signal, the Zn-NEXFS (zinc near-edge X-ray fluorescence spectroscopy) data were collected. Zn-NEXFS differs from Zn-XANES in the signal detection, in that it uses the fluorescence signal to obtain an absorption signal which is inversely proportional to the XANES signal. The fluorescence detector only measures the signal from the interactions of the beam with the zinc atoms. In contrast, in the ion chamber used for XANES measurements an extremely small percentage of the incoming radiation reaching the ion chamber is absorbed by Zn atoms. Thus the absorption signal is extremely small relative to the large amount of radiation reaching the detector. Use of the fluorescence detector allows for a signal with minimal background. This is undoubtedly why fluorescence is the method most frequently employed for natural samples. The samples were analyzed three to five times with a dwell time ranging from 0.5 to 3 s per step, with higher dwell times given to samples with low concentrations. Care was taken to minimize detector saturation and the dead time (<10%), while optimizing the signal, by adjusting the detector position and the size of the slits, in accordance with methods to optimize the quality of analysis conducted using fluorescence in X-ray absorption spectroscopy (XAS) analysis as discussed in the literature (Abe *et al.*, 2018 ▸; Gräfe *et al.*, 2014 ▸; Waychunas *et al.*, 2003 ▸).

The spectra were analyzed with *ATHENA* XAS data processing software (*Demeter* 0.9.26 using *Ifeffit* 1.2.12) (Ravel & Newville, 2005 ▸). The X-ray fluorescence signal was normalized by dividing the zinc counts by the upstream ion chamber counts. Since each sample had been scanned multiple times, these scans were aligned within *ATHENA* based on the smoothed derivative of energy before merging the absorption edge, with importance placed on larger edge steps, in order to assign higher importance to spectra obtained from a more concentrated sample. Edge-step positions were determined by using the first derivative of the spectra.

All XANES data is provided in Table S1 of the supporting information.

The identity of the mineral samples was verified on a Malvern PANalytical Empyrean multi-technique X-ray diffractometer and a copper radiation source. Data analysis was conducted with the *HighScore Plus* software associated with the diffractometer, and a third-party database, PDF 4+, from the International Centre for Diffraction Data (ICDD). Each sample was approximately a gram in size and was placed on a silica zero plate to minimize background signals from the sample mount. The masks and slits on the Empyrean X-ray diffractometer were set for the small sample size and each mineral was analyzed for 10–40 s per step, with a 2Θ range from 10–90°. Many of the samples were analyzed while spinning at 3.755 rpm, to minimize diffraction errors. XRD data are provided in the supporting information.

## 3. Results and discussion

The XANES spectra of all zinc materials in this study had principal *K*-edge peak energies between 9660.5 and 9666.0 eV. This peak results from the excitation of an electron from a 1*s* inner orbital to higher-energy orbitals as a result of interaction with a synchrotron-generated X-ray. Subsequent decay of higher-energy electrons to unoccupied 1*s* orbitals releases photons, which are counted by the Vortex-EM detector. The minerals and organics measured in this study contained zinc in the +II oxidation state. Although the structural position of zinc within the mineral lattice would be expected to exert a major influence on spectral features, a study investigating the impact of tetrahedral versus octahedral coordination on spectral features shows that these changes are subtle (Waychunas *et al.*, 2003 ▸).

Spectra of most minerals also revealed additional pre-edge or post-edge features that can be used to identify specific minerals or differentiate between mineral groups. In general, these pre- and post-edge features are related to (i) the presence and weight percent of different elements; (ii) the oxidation state of these elements; and (iii) the arrangement of these elements in the mineral structure. More information on pre-edge and post-edge features for specific minerals and mineral groups follows.

### 3.1. Zinc silicates

**Figure 1:**
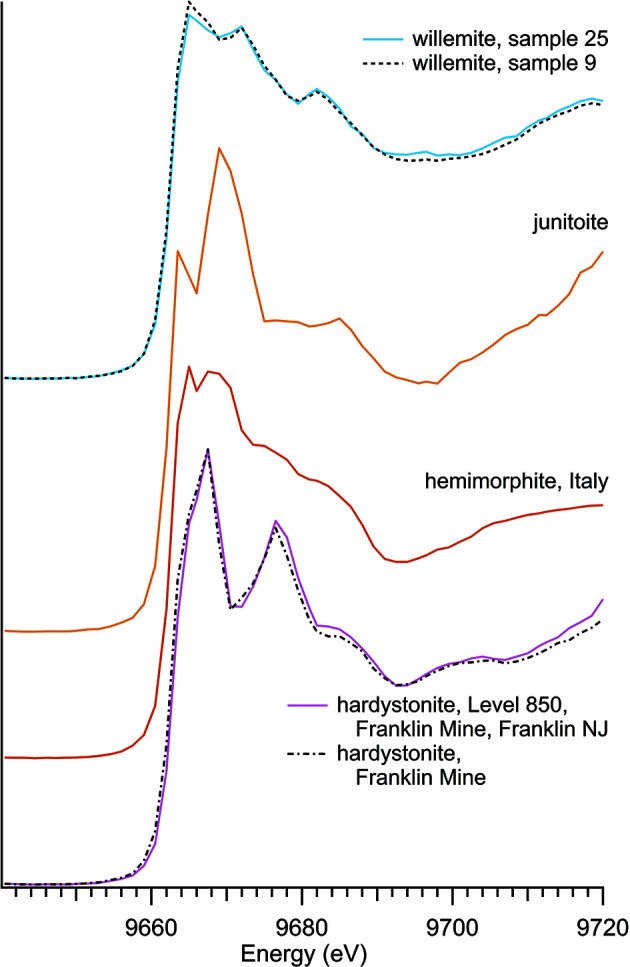
Zinc silicate spectra.

**Figure 2:**
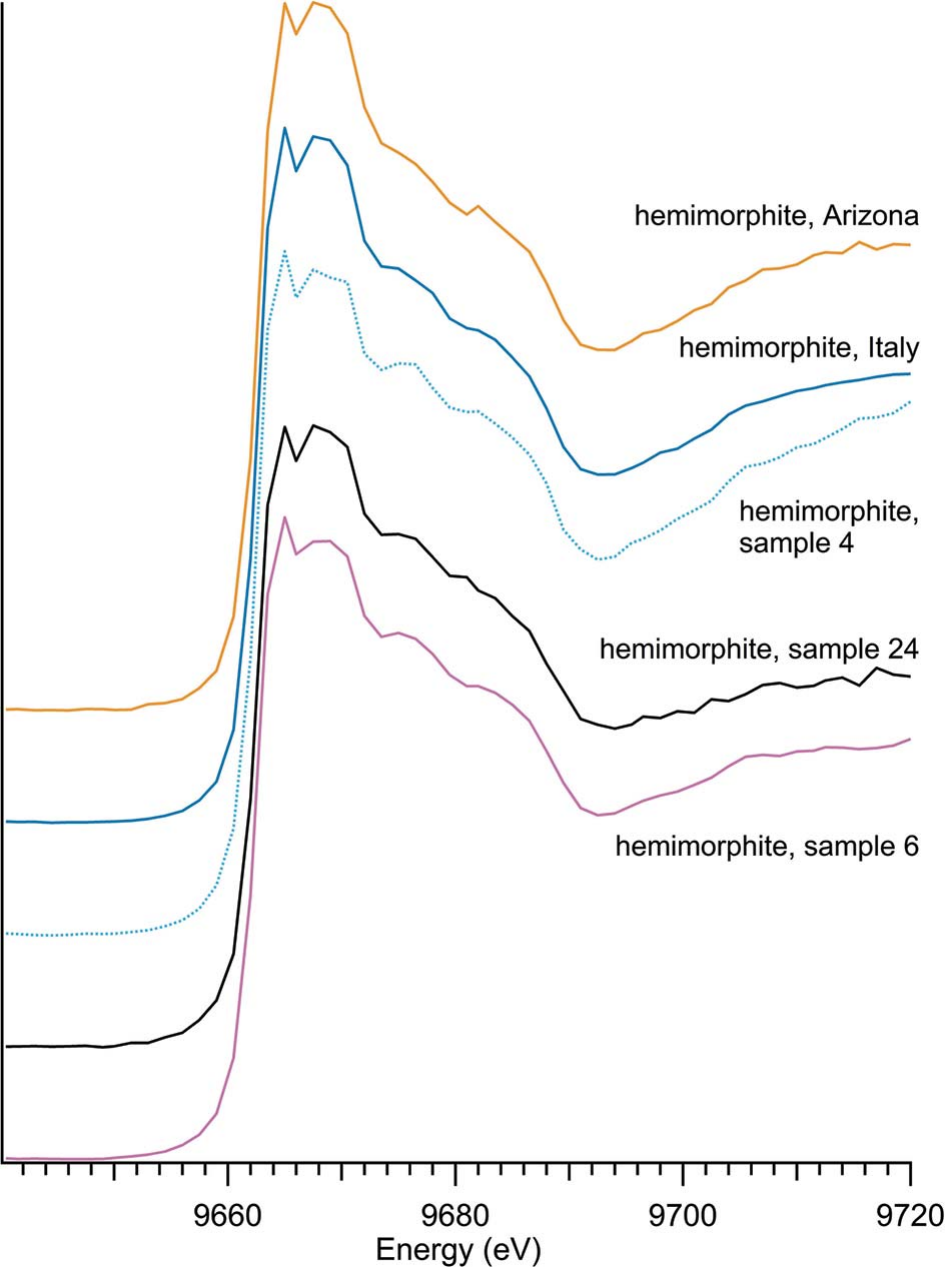
Comparison of hemimorphite minerals.

Four unique zinc silicate minerals were studied including three sorosilicates – hardystonite, hemimorphite and junitoite – and one nesosilicate – willemite (Table 1 ▸), with spectra shown in Fig. 1 ▸. The analysis includes five separate samples of the zinc silicate hemimorphite (Fig. 2 ▸), to assess spectroscopic variation across natural samples from different locations. The absorption edge position of 9663.5 eV was the same for all zinc silicates regardless of composition. Zinc silicate spectra all have two distinctive peaks at post-edge energies, the strength of which appears to be unique to this mineral class. Sorosilicate versus nesosilicate arrangement of the Si tetrahedrons does not appear to be the primary control of spectral features as the three sorosilicates each have post-edge peaks at different energies (junitoite at 9669 eV, hardystonite at 9676 eV and a broad hemimorphite secondary peak around 9668 eV).

### 3.2. Zinc carbonates

**Figure 3:**
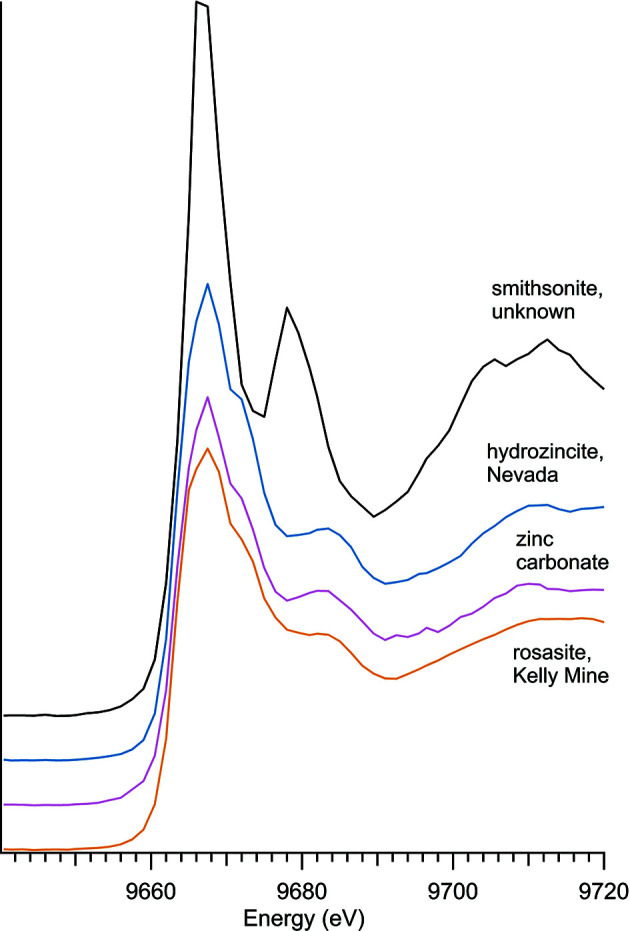
Zinc carbonate spectra.

Three natural zinc carbonate minerals – hydro­zincite, rosasite and smithsonite – were observed alongside a commercial basic zinc carbonate (Table 2 ▸) with spectra shown in Fig. 3 ▸. The edge positions for the hydrous forms – hydro­zincite, rosasite and basic zinc carbonate – are all at 9663.5 eV, and the anhydrous smithsonite edge is at 9666.0 eV. The spectra of the hydrous zinc carbonates are all characterized by a wider main peak with a prominent shoulder and a higher-energy peak at approximately 9681 eV. Smithsonite differs from the hydrous zinc carbonates with a narrower main peak without a prominent shoulder, a very strong higher-energy peak at 9678 eV, and a wider peak around 9710 eV. These results match well with previous analysis of smithsonite, including those measured in transmission mode (Gao *et al.*, 2014 ▸) and in fluorescence mode (Bazin *et al.*, 2009 ▸).

### 3.3. Zinc phosphates

**Figure 4:**
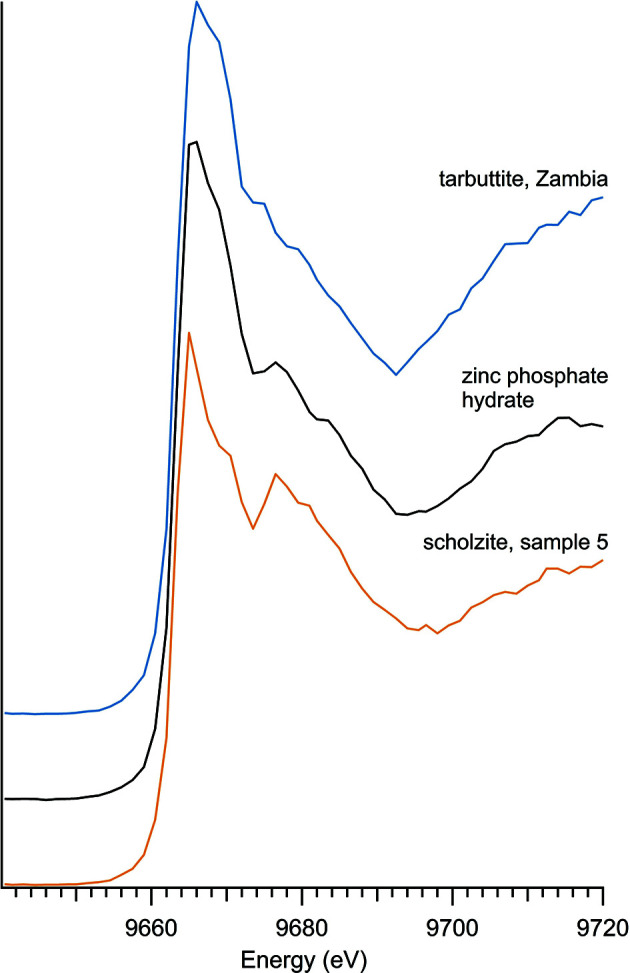
Zinc phosphate spectra.

Zinc phosphates are distinguishable from the larger selection of oxyanions because of the similarity of their spectra (Fig. 4 ▸) and the consistency of the initial *K*-edge peak energy of 9663.5 eV (Table 3 ▸). The zinc phosphates contained within this study are the natural minerals scholzite and tarbuttite and a synthetic zinc phosphate hydrate. Zinc phosphate spectra are characterized by a wide main peak which is likely due to the presence of a higher-energy peak at 9670 eV. All of the zinc phosphate spectra have a secondary peak at approximately 9676.5 eV which varies in intensity in the different zinc phosphates.

### 3.4. Other zinc minerals and compounds with zinc oxyanions

**Figure 5:**
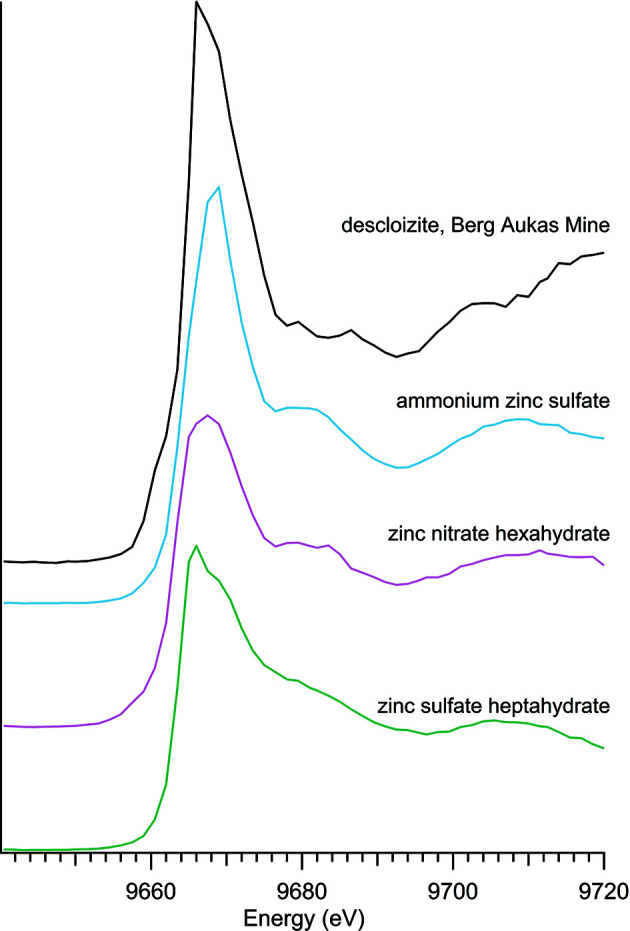
Zinc oxyanion spectra.

**Figure 6:**
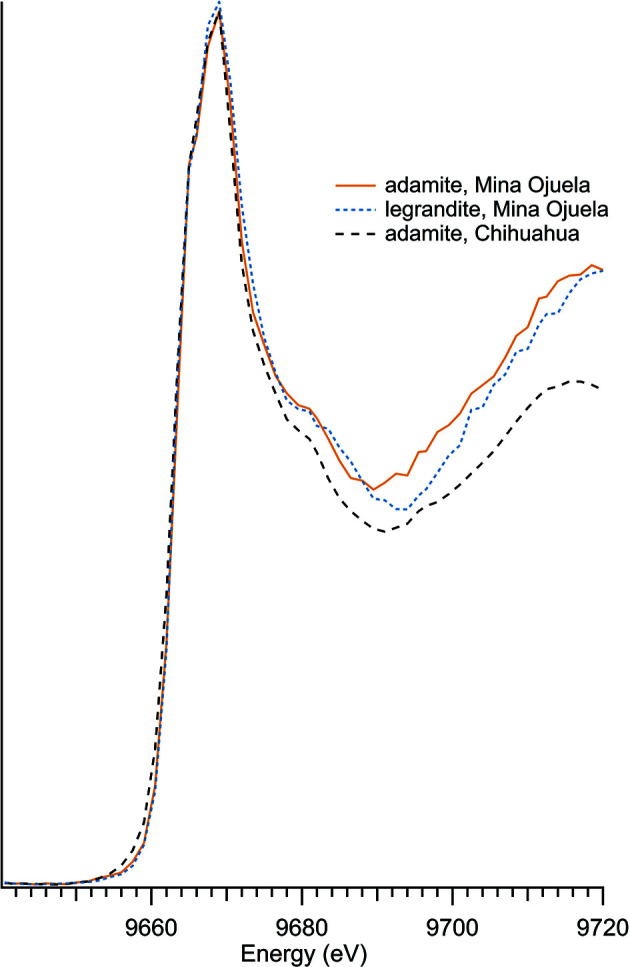
Zinc arsenate spectra.

Zinc compounds with oxyanions other than phosphate were investigated (Table 4 ▸), with spectra shown in Fig. 5 ▸. Zinc arsenates are shown separately in Fig. 6 ▸ to further illustrate the reproducibility of XANES spectra, regardless of sample location. The zinc arsenates have a nearly identical edge shape and intensity, with divergence only occurring post-edge, around 9680 eV.

### 3.5. Zinc oxides

**Figure 7:**
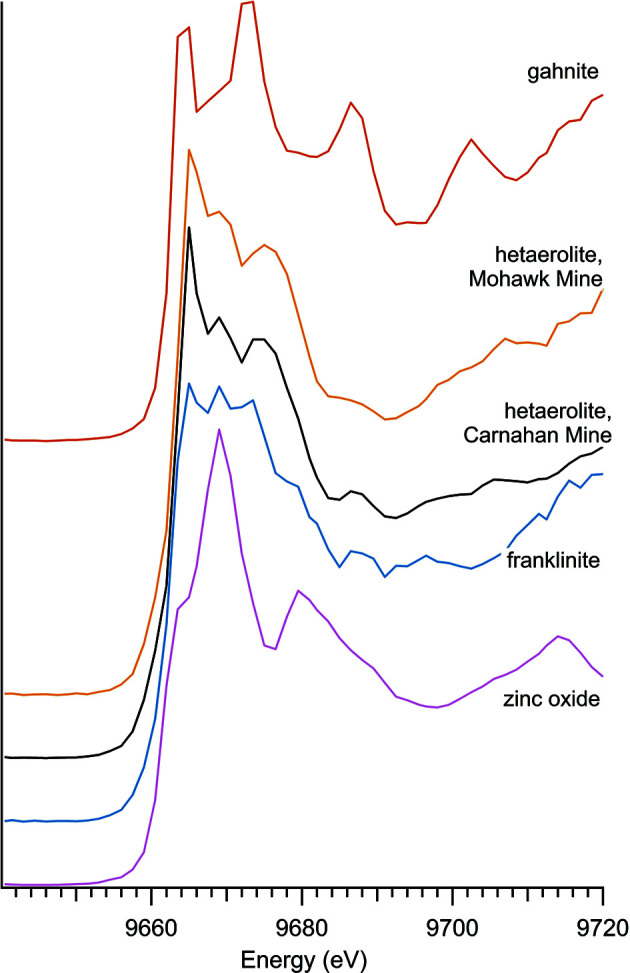
Spectra of zinc oxides.

There were four zinc oxide materials used in this study: three naturally occurring minerals – franklinite, gahnite and hetaerolite – and a synthetic zinc oxide compound (Table 5 ▸ and Fig. 7 ▸).

**Figure 8:**
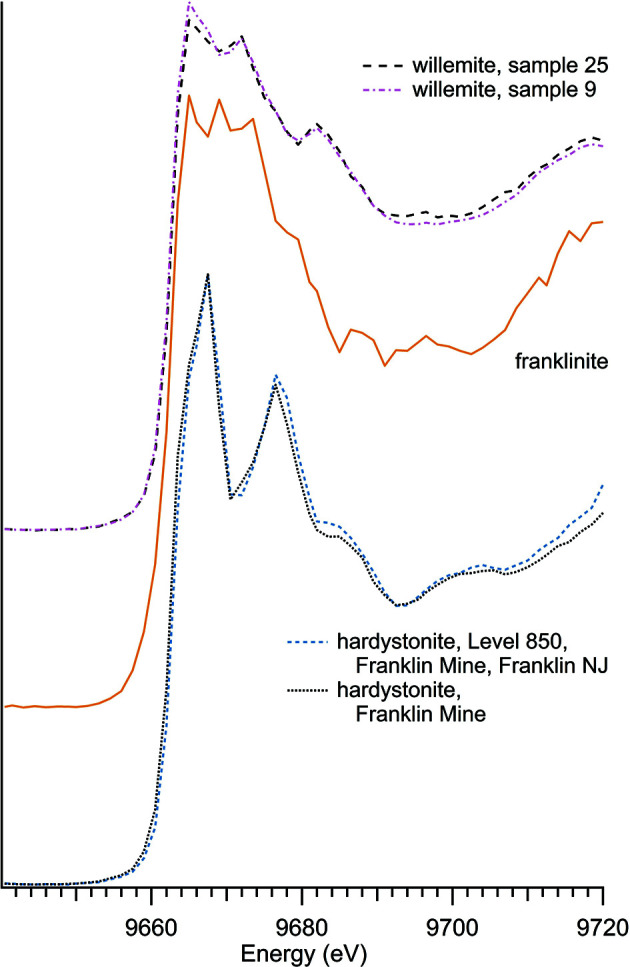
Comparison of franklinite specra with zinc silicates.

The spectra for the naturally occurring minerals do not resemble the spectra for zinc oxide. Franklinite more closely matches the spectra for hardystonite and willemite, minerals with which franklinite is closely associated in natural environments. A comparison of these spectra is shown in Fig. 8 ▸. Although franklinite, dominated by three strong peaks, appears aberrant, it matches well with previously run franklinite Zn *K*-edge spectra (Waychunas *et al.*, 2003 ▸; Hamilton *et al.*, 2016 ▸).

There were two samples of hetaerolite, but due to the difficulty of extraction of this mineral there was not enough of the sample from Mohawk Mine to allow for independent verification using the X-ray diffractometer. However, given the similarities of the XAS spectra, and the identical *K*-edge energy, there is a high level of confidence in the identification of the hetaerolite sample from Mohawk Mine.

The zinc oxide sample had one of the lowest observed *K*-edge values. However, the unique shape and low energy level of the zinc *K*-edge may have implications for positively identifying anthropogenic zinc oxides using XANES (Hamilton *et al.*, 2016 ▸).

### 3.6. Organic zinc compounds

**Figure 9:**
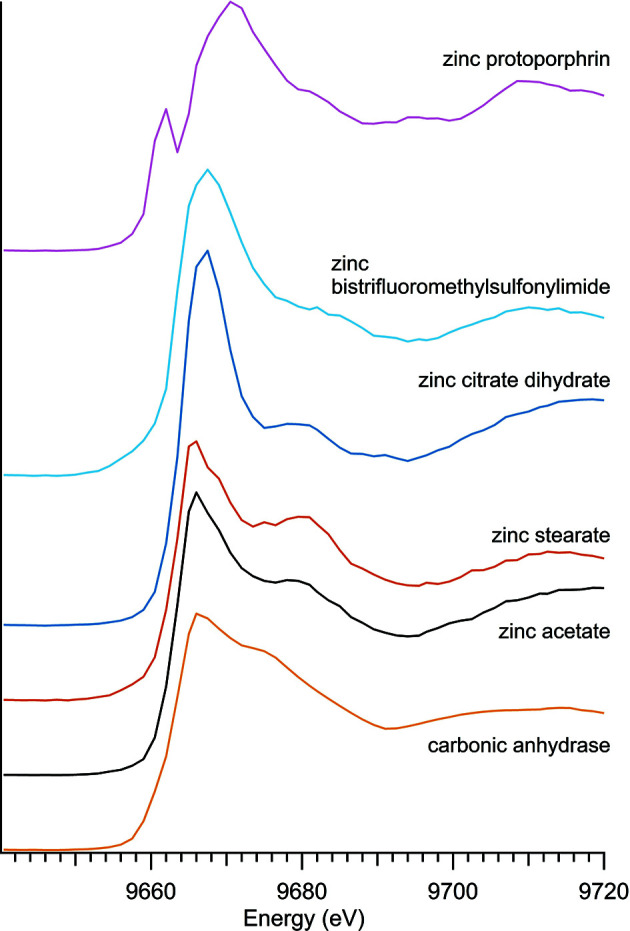
Spectra of organic zinc.

The six organic zinc compounds (Table 6 ▸) shared a general spectral shape (Fig. 9 ▸), with the significant exception of zinc protoporphyrin, but their peak energy varied more than the other subgroups represented here. For zinc acetate and zinc stearate, the energy peak was shifted to 9665 eV, as illustrated in Table 6 ▸. The organic zinc samples had a wide main peak, which is most readily observed with the carbonic anhydrase.

The peaks around the *K*-edge of zinc protoporphyrin were closely aligned across all four synchrotron runs; however, the position and intensity of subtle peaks at energies above the *K*-edge were somewhat variable between synchrotron runs for this material.

### 3.7. Additional zinc compounds

**Figure 10:**
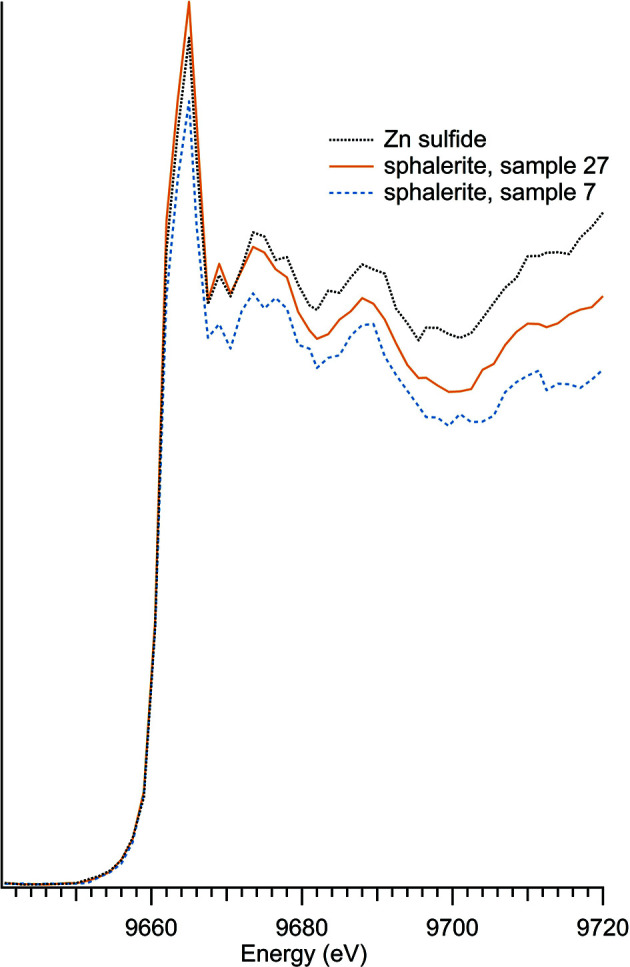
Zinc sulfur spectra.

**Figure 11:**
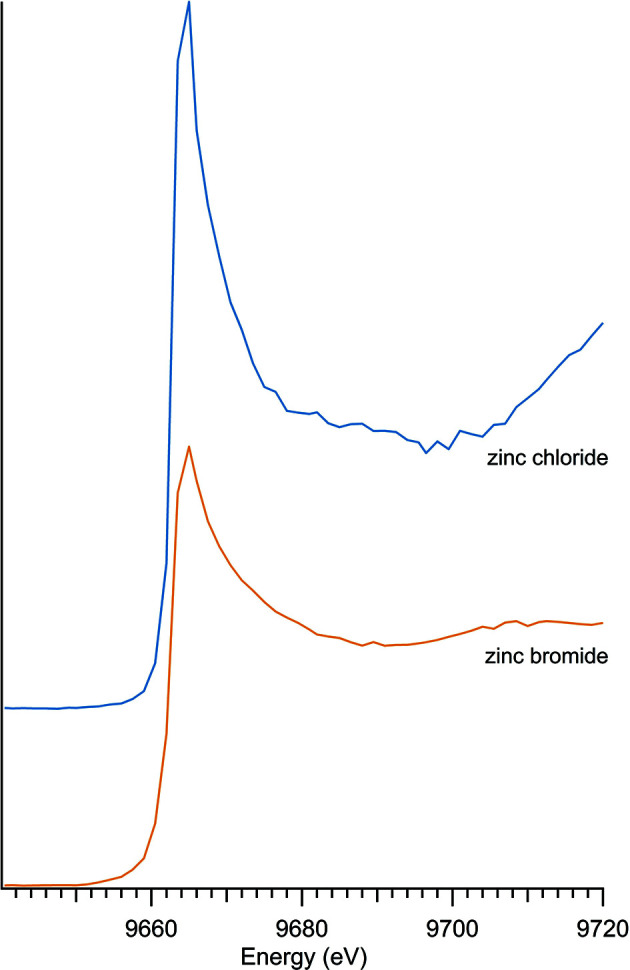
Zinc halogen spectra.

Other zinc compounds, which did not fit into the previous categories, include zinc halogens and zinc sulfides (Table 7 ▸). There were three samples which contained zinc and sulfur, shown in Fig. 10 ▸, and two halogen samples, which included zinc bromide and zinc chloride, illustrated in Fig. 11 ▸.

Sphalerite has a strong edge and closely aligned peak, but the sphalerite samples were consistently noisy on the post-edge, past about 5 eV from the *K*-edge peak energy. While the presence of iron, visually apparent through the black color of the sphalerite (Hurlbut, 1941 ▸), could impact the spectral shape, both sphalerite samples were almost colorless, indicating a very low iron content. This assessment is further informed by observing the spectra of sphalerite, which is closely matched to the synthetic zinc sulfide.

Both zinc bromide and zinc chloride exhibited a strong peak and a nearly featureless post-edge.

## 4. Conclusions

The consistency of spectral patterns between specimens of the same mineral from different localities suggests that natural compositional variations will not influence identification of these phases in unknown samples. The variety of distinctive spectra features for the minerals, compounds and organic species will aid in the identification of pure forms of these species and will also be helpful in the application of spectral linear combination approaches for the identification of unknowns in mixtures.

## Supplementary Material

XANES and XRD data (.xlsx format). DOI: 10.1107/S160057751900540X/rv5107sup1.xlsx

XANES and XRD data (.pdf format). DOI: 10.1107/S160057751900540X/rv5107sup2.pdf

## Figures and Tables

**Table 1:** Zinc silicates

Sample number	Name	Ideal chemical formula	Locality	*E*_0_ (eV)
32	Hardystonite	Ca_2_Zn(Si_2_O_7_)	850 Level, Hamburg Mine, Franklin, NJ, USA	9663.5
40	Hardystonite	Ca_2_Zn(Si_2_O_7_)	Franklin, NJ, USA	9663.5
4	Hemimorphite	Zn_4_Si_2_O_7_(OH)_2_·H_2_O	Private collection, unknown locality	9663.5
6	Hemimorphite	Zn_4_Si_2_O_7_(OH)_2_·H_2_O	Private collection, unknown locality	9663.5
24	Hemimorphite	Zn_4_Si_2_O_7_(OH)_2_·H_2_O	Private collection, unknown locality	9663.5
36	Hemimorphite	Zn_4_Si_2_O_7_(OH)_2_·H_2_O	79 Mine, Banner District, Gila Co., AZ, USA	9663.5
37	Hemimorphite	Zn_4_Si_2_O_7_(OH)_2_·H_2_O	Sardinia, Italy	9663.5
31	Junitoite	CaZn_2_Si_2_O_7_·H_2_O	Christmas Mine, Gila County, AZ, USA	9663.5
25	Willemite	Zn_2_SiO_4_	Private collection, unknown locality	9663.5
9	Willemite	Zn_2_SiO_4_	Private collection, unknown locality	9663.5

**Table 2:** Zinc carbonates

Sample number	Name	Ideal chemical formula	Locality	*E*_0_ (eV)
41	Hydro­zincite	Zn_5_(CO_3_)_2_(OH)_6_	Goodsprings, NV, USA	9663.5
35	Rosasite	(Cu,Zn)_2_(CO_3_)(OH)_2_	Kelly Mine, Magdalena, NM, USA	9663.5
26	Smithsonite	ZnCO_3_	Private collection, unknown location	9666.0
N/A	Zinc carbonate	Zn_5_(CO_3_)_2_·(OH)_6_	Alfa A14590	9663.5

**Table 3:** Zinc phosphates

Sample number	Name	Ideal chemical formula	Locality	*E*_0_ (eV)
5	Scholzite	CaZn_2_(PO_4_)_2_·2H_2_O	Private collection, unknown location	9663.5
8	Tarbuttite	Zn_2_(PO_4_)(OH)	Zambia Broken Hill Mine	9663.5
17	Zinc phosphate hydrate	Zn_3_(PO_4_)xH_2_O	Alfa 11589	9663.5

**Table 4:** Additional zinc oxyanions

Sample number	Name	Ideal chemical formula	Locality	*E*_0_ (eV)
30	Adamite	Zn_2_(AsO_4_)(OH)	Mina Ojuela, Mapimi, Durango, Mexico	9663.5
39	Adamite	Zn_2_(AsO_4_)(OH)	Chihuahua, Mexico	9663.5
1	Ammonium zinc sulfate hydrate	(NH_4_)_2_Zn(SO_4_)_2_·H_2_O	Alfa A13584	9665.0
29	Descloizite	PbZn(VO_4_)(OH)	Berg Aukas Mine, Grootfontein District, Namibia	9666.0
28	Legrandite	Zn_2_(AsO_4_)(OH)·H_2_O	Mina Ojuela, Mapimi, Durango, Mexico	9663.5
15	Zinc nitrate hexahydrate	Zn(NO_3_)_2_·6H_2_O	Acros Organics 211660050	9663.5
19	Zinc sulfate heptahydrate	(SO_4_)Zn·7H_2_O	Alfa 33399	9665.0

**Table 5:** Zinc oxides

Sample number	Name	Ideal chemical formula	Locality	*E*_0_ (eV)
22	Franklinite	ZnFe_2_O_4_	Franklin, NJ, USA, private collection of Ellery Ingall	9663.5
33	Gahnite	ZnAl_2_O_4_	Falun Mine, Falun, Dalarna, Sweden	9663.5
34	Hetaerolite	ZnMn_2_O_4_	Mohawk Mine, San Bernardino County, CA, USA	9665.0
38	Hetaerolite	ZnMn_2_O_4_	Carnahan Mine, Golden, NM, USA	9665.0
16	Zinc oxide	ZnO	CAS 1314-13-2	9662.0

**Table 6:** Organic zinc samples

Sample number	Name	Ideal chemical formula	Locality	*E*_0_ (eV)
N/A	Carbonic anhydrase		MP Biomedicals 153879	9665.0
10	Zinc acetate	Zn(CH_3_COO)_2_·2H_2_O	EM CAS5970-45-6	9665.0
20	Zinc bis (tri­fluoro­methyl­sulfonyl)­imide	C_4_F_12_N_2_O_8_S_4_Zn	Alfa Aesar (chemical formula from #46880)	9663.5
14	Zinc citrate dihydrate	(C_6_H_5_O_7_)_2_Zn_3_·2H_2_O	Aldrich 480762	9663.5
N/A	Zinc protoporphyrin, 96%	C_34_H_32_N_4_O_4_Zn	Alfa Aesar	9660.5
18	Zinc stearate	C_36_H_70_O_4_Zn	Alfa 33238	9665.0

**Table 7:** Additional zinc compounds

Sample number	Name	Ideal chemical formula	Locality	*E*_0_ (eV)
7	Sphalerite	ZnS	Private collection, unknown location	9662.0
27	Sphalerite	ZnS	Private collection, unknown location	9662.0
11	Zinc bromide	ZnBr_2_	Acros A0356300	9664.0
13	Zinc chloride	ZnCl_2_	Alfa A16281	9665.0
23	Zinc sulfide	ZnS	Alfa Aesar 40091	9662.0
