# Supplementary material for: Zinc K-edge XANES spectroscopy of mineral and organic standards
Source: J Synchrotron Radiat. 2019 Jun 21;26(Pt 4):1302–9. doi: 10.1107/S160057751900540X (PMC6613124; doi:10.1107/S160057751900540X)
Supplement: Supplementary file 2 [file s-26-01302-sup2.pdf]

JOURNAL OF SYNCHROTRON RADIATION

Volume 26 (2019)

Supporting information for article:

Zinc *K*-edge XANES spectroscopy of mineral and organic standards

Erin K. Griffith, Ellery D. Ingall, Peter L. Morton, David A. Tavakoli and Barry Lai

Table S1 Zinc XANES Spectral Energy Reference Standards

energy (eV)	adamite 30	adamite 39	ammonium Zn sulfate	brass alloy	carbonic anhydrase	descloizite 29	franklinite 22
9640.5	1.69E-03	1.98E-03	8.75E-04	3.05E-03	1.13E-03	3.55E-03	-2.63E-04
9641.5	4.47E-05	1.01E-06	1.60E-03	2.51E-03	2.67E-04	2.34E-05	3.85E-03
9643.0	1.21E-04	4.48E-05	-1.23E-03	-3.57E-03	-7.19E-04	-2.06E-03	-2.11E-03
9644.5	-2.59E-03	-1.63E-03	-1.03E-03	-2.94E-03	2.62E-04	7.28E-04	1.63E-03
9646.0	6.78E-04	-2.18E-03	-6.96E-04	1.29E-04	-1.25E-03	-3.58E-03	-1.32E-03
9647.5	-1.22E-03	-3.47E-03	-8.55E-04	-3.43E-03	-1.24E-03	-4.91E-03	5.96E-04
9649.0	-1.74E-03	2.68E-04	1.56E-03	-2.99E-03	2.22E-04	3.61E-03	4.49E-04
9650.0	3.06E-03	2.47E-03	3.30E-04	7.64E-03	2.62E-03	2.76E-03	-5.07E-04
9651.5	6.17E-03	6.57E-03	1.40E-03	1.14E-02	3.71E-03	5.50E-03	2.36E-03
9653.0	1.77E-02	1.59E-02	7.03E-03	2.03E-02	9.57E-03	1.57E-02	9.33E-03
9654.5	2.47E-02	3.12E-02	1.60E-02	4.73E-02	1.81E-02	2.71E-02	2.31E-02
9656.0	3.49E-02	5.66E-02	2.79E-02	1.08E-01	3.43E-02	4.84E-02	4.44E-02
9657.5	6.78E-02	1.03E-01	5.87E-02	2.30E-01	7.50E-02	9.18E-02	1.03E-01
9659.0	1.26E-01	1.90E-01	1.23E-01	4.44E-01	1.92E-01	2.49E-01	2.12E-01
9660.5	3.08E-01	4.23E-01	2.16E-01	5.38E-01	3.90E-01	5.56E-01	4.03E-01
9662.0	7.54E-01	9.04E-01	4.18E-01	6.32E-01	6.23E-01	7.66E-01	7.74E-01
9663.5	1.51E+00	1.63E+00	9.52E-01	7.57E-01	1.02E+00	1.17E+00	1.42E+00
9665.0	2.25E+00	2.25E+00	1.62E+00	8.97E-01	1.44E+00	2.31E+00	1.72E+00
9666.0	2.34E+00	2.40E+00	1.96E+00	9.52E-01	1.58E+00	3.41E+00	1.65E+00
9667.5	2.62E+00	2.64E+00	2.44E+00	1.04E+00	1.54E+00	3.27E+00	1.61E+00
9669.0	2.73E+00	2.73E+00	2.53E+00	1.11E+00	1.48E+00	3.11E+00	1.71E+00
9670.5	2.44E+00	2.35E+00	2.08E+00	1.06E+00	1.41E+00	2.69E+00	1.63E+00
9672.0	2.01E+00	1.94E+00	1.71E+00	9.81E-01	1.37E+00	2.36E+00	1.63E+00
9673.5	1.79E+00	1.73E+00	1.43E+00	9.46E-01	1.35E+00	2.06E+00	1.66E+00
9675.0	1.69E+00	1.62E+00	1.23E+00	8.94E-01	1.33E+00	1.74E+00	1.52E+00
9676.5	1.59E+00	1.53E+00	1.17E+00	8.91E-01	1.28E+00	1.50E+00	1.37E+00
9678.0	1.53E+00	1.45E+00	1.19E+00	9.04E-01	1.22E+00	1.44E+00	1.34E+00
9679.5	1.50E+00	1.42E+00	1.19E+00	9.01E-01	1.15E+00	1.46E+00	1.32E+00
9681.0	1.49E+00	1.39E+00	1.18E+00	8.99E-01	1.10E+00	1.41E+00	1.20E+00
9682.0	1.46E+00	1.35E+00	1.18E+00	8.76E-01	1.06E+00	1.37E+00	1.17E+00
9683.5	1.39E+00	1.27E+00	1.13E+00	8.17E-01	1.01E+00	1.36E+00	1.07E+00
9685.0	1.32E+00	1.21E+00	1.06E+00	8.01E-01	9.61E-01	1.38E+00	1.00E+00
9686.5	1.27E+00	1.17E+00	9.95E-01	7.56E-01	9.14E-01	1.41E+00	1.06E+00
9688.0	1.26E+00	1.14E+00	9.31E-01	7.57E-01	8.71E-01	1.35E+00	1.05E+00
9689.5	1.23E+00	1.11E+00	8.78E-01	7.57E-01	8.34E-01	1.32E+00	1.03E+00
9691.0	1.26E+00	1.10E+00	8.46E-01	7.48E-01	8.08E-01	1.27E+00	9.62E-01
9692.5	1.28E+00	1.11E+00	8.24E-01	7.62E-01	8.11E-01	1.25E+00	1.01E+00
9694.0	1.28E+00	1.13E+00	8.26E-01	7.74E-01	8.24E-01	1.27E+00	1.00E+00
9695.5	1.35E+00	1.17E+00	8.53E-01	7.63E-01	8.41E-01	1.28E+00	1.03E+00
9696.5	1.35E+00	1.18E+00	8.75E-01	7.66E-01	8.54E-01	1.33E+00	1.05E+00
9698.0	1.41E+00	1.19E+00	9.08E-01	7.79E-01	8.71E-01	1.40E+00	1.02E+00
9699.5	1.44E+00	1.22E+00	9.56E-01	7.86E-01	8.86E-01	1.46E+00	1.02E+00
9701.0	1.47E+00	1.25E+00	9.99E-01	8.04E-01	9.02E-01	1.53E+00	1.00E+00
9702.5	1.53E+00	1.28E+00	1.03E+00	7.71E-01	9.12E-01	1.57E+00	9.95E-01
9704.0	1.56E+00	1.32E+00	1.07E+00	7.61E-01	9.22E-01	1.57E+00	1.01E+00
9705.5	1.59E+00	1.35E+00	1.09E+00	7.39E-01	9.28E-01	1.57E+00	1.04E+00
9707.0	1.65E+00	1.40E+00	1.11E+00	7.19E-01	9.32E-01	1.55E+00	1.06E+00
9708.5	1.72E+00	1.45E+00	1.12E+00	6.87E-01	9.33E-01	1.62E+00	1.12E+00
9710.0	1.74E+00	1.49E+00	1.12E+00	6.85E-01	9.36E-01	1.61E+00	1.17E+00
9711.5	1.83E+00	1.53E+00	1.09E+00	6.54E-01	9.37E-01	1.70E+00	1.21E+00
9712.5	1.84E+00	1.55E+00	1.09E+00	6.64E-01	9.44E-01	1.72E+00	1.19E+00
9714.0	1.89E+00	1.56E+00	1.08E+00	6.38E-01	9.48E-01	1.82E+00	1.28E+00
9715.5	1.90E+00	1.57E+00	1.04E+00	6.41E-01	9.47E-01	1.81E+00	1.34E+00
9717.0	1.91E+00	1.57E+00	1.03E+00	6.22E-01	9.32E-01	1.86E+00	1.31E+00
9718.5	1.94E+00	1.56E+00	1.01E+00	6.05E-01	9.25E-01	1.87E+00	1.36E+00
9720.0	1.92E+00	1.55E+00	1.00E+00	5.97E-01	9.13E-01	1.88E+00	1.37E+00

energy (eV)	gahnite 33	hardy stonite 32	hardy stonite 40	hemi morphite 4	hemi morphite 6	hemi morphite 24	hemi morphite 36	hemi morphite 37
9640.5	2.72E-03	2.13E-03	1.51E-03	1.72E-03	7.28E-04	-2.91E-04	4.22E-04	1.05E-03
9641.5	-2.55E-04	1.05E-03	1.35E-04	6.05E-04	-4.97E-04	-6.14E-05	1.20E-03	4.48E-05
9643.0	-9.98E-04	-6.27E-04	-5.90E-04	-1.76E-03	2.51E-04	5.58E-04	-1.28E-03	3.44E-04
9644.5	-9.26E-04	-1.29E-03	-1.57E-03	-2.42E-03	-6.12E-04	-3.51E-04	-7.52E-04	-1.77E-03
9646.0	-2.67E-03	-2.51E-04	-3.35E-04	-1.51E-03	-9.09E-04	1.94E-04	-1.34E-03	-5.36E-04
9647.5	-5.91E-04	-2.66E-04	-8.73E-04	1.14E-03	-9.08E-04	1.37E-03	1.49E-03	-6.14E-04
9649.0	1.29E-03	-1.62E-03	5.78E-04	5.90E-04	-6.01E-04	-1.58E-03	1.15E-03	5.29E-04
9650.0	1.50E-03	1.69E-03	1.24E-03	1.09E-03	1.99E-03	2.71E-04	-4.85E-04	9.50E-04
9651.5	4.68E-03	3.08E-03	4.12E-03	4.59E-03	5.05E-03	7.97E-03	1.93E-04	2.94E-03
9653.0	8.59E-03	5.07E-03	7.41E-03	8.87E-03	9.22E-03	7.96E-03	1.24E-02	7.01E-03
9654.5	1.33E-02	1.31E-02	1.64E-02	1.77E-02	1.60E-02	2.04E-02	1.47E-02	1.38E-02
9656.0	2.48E-02	2.07E-02	2.50E-02	3.10E-02	3.02E-02	3.04E-02	2.37E-02	2.47E-02
9657.5	4.71E-02	3.47E-02	4.30E-02	5.71E-02	5.35E-02	5.90E-02	4.80E-02	4.82E-02
9659.0	8.41E-02	7.28E-02	9.20E-02	1.09E-01	1.00E-01	1.03E-01	8.68E-02	8.98E-02
9660.5	2.07E-01	1.60E-01	2.11E-01	2.36E-01	2.25E-01	2.06E-01	2.07E-01	2.05E-01
9662.0	5.79E-01	4.47E-01	5.50E-01	6.16E-01	5.87E-01	5.50E-01	5.58E-01	5.87E-01
9663.5	1.59E+00	1.06E+00	1.21E+00	1.35E+00	1.26E+00	1.21E+00	1.29E+00	1.32E+00
9665.0	1.63E+00	1.43E+00	1.46E+00	1.52E+00	1.43E+00	1.38E+00	1.57E+00	1.55E+00
9666.0	1.29E+00	1.52E+00	1.56E+00	1.42E+00	1.35E+00	1.30E+00	1.50E+00	1.45E+00
9667.5	1.34E+00	1.72E+00	1.72E+00	1.48E+00	1.37E+00	1.38E+00	1.58E+00	1.53E+00
9669.0	1.38E+00	1.42E+00	1.34E+00	1.46E+00	1.38E+00	1.37E+00	1.56E+00	1.52E+00
9670.5	1.42E+00	1.10E+00	1.08E+00	1.45E+00	1.34E+00	1.34E+00	1.51E+00	1.46E+00
9672.0	1.72E+00	1.10E+00	1.13E+00	1.29E+00	1.21E+00	1.19E+00	1.34E+00	1.29E+00
9673.5	1.73E+00	1.18E+00	1.19E+00	1.26E+00	1.16E+00	1.14E+00	1.26E+00	1.24E+00
9675.0	1.41E+00	1.29E+00	1.29E+00	1.27E+00	1.17E+00	1.14E+00	1.24E+00	1.23E+00
9676.5	1.23E+00	1.44E+00	1.41E+00	1.27E+00	1.16E+00	1.13E+00	1.22E+00	1.21E+00
9678.0	1.14E+00	1.37E+00	1.30E+00	1.22E+00	1.12E+00	1.09E+00	1.18E+00	1.18E+00
9679.5	1.13E+00	1.21E+00	1.16E+00	1.17E+00	1.08E+00	1.05E+00	1.13E+00	1.12E+00
9681.0	1.12E+00	1.09E+00	1.04E+00	1.16E+00	1.05E+00	1.05E+00	1.10E+00	1.10E+00
9682.0	1.12E+00	1.02E+00	9.99E-01	1.16E+00	1.05E+00	1.02E+00	1.12E+00	1.10E+00
9683.5	1.14E+00	1.02E+00	9.80E-01	1.14E+00	1.04E+00	9.98E-01	1.09E+00	1.07E+00
9685.0	1.22E+00	1.01E+00	9.80E-01	1.11E+00	1.01E+00	9.59E-01	1.05E+00	1.03E+00
9686.5	1.33E+00	9.76E-01	9.53E-01	1.07E+00	9.75E-01	9.25E-01	1.02E+00	9.92E-01
9688.0	1.27E+00	9.32E-01	9.21E-01	1.00E+00	9.08E-01	8.52E-01	9.50E-01	9.21E-01
9689.5	1.06E+00	8.81E-01	8.53E-01	9.01E-01	8.37E-01	7.92E-01	8.68E-01	8.35E-01
9691.0	9.05E-01	8.23E-01	8.14E-01	8.57E-01	7.81E-01	7.29E-01	8.13E-01	7.86E-01
9692.5	8.50E-01	7.85E-01	7.86E-01	8.34E-01	7.65E-01	7.17E-01	8.02E-01	7.74E-01
9694.0	8.59E-01	7.86E-01	7.91E-01	8.41E-01	7.69E-01	7.08E-01	8.01E-01	7.74E-01
9695.5	8.56E-01	8.11E-01	8.03E-01	8.72E-01	7.86E-01	7.17E-01	8.21E-01	7.89E-01
9696.5	8.57E-01	8.30E-01	8.23E-01	8.81E-01	7.97E-01	7.35E-01	8.38E-01	8.00E-01
9698.0	9.26E-01	8.55E-01	8.44E-01	8.99E-01	8.09E-01	7.31E-01	8.46E-01	8.23E-01
9699.5	1.03E+00	8.71E-01	8.62E-01	9.25E-01	8.17E-01	7.47E-01	8.66E-01	8.31E-01
9701.0	1.12E+00	8.77E-01	8.75E-01	9.44E-01	8.32E-01	7.43E-01	8.90E-01	8.54E-01
9702.5	1.19E+00	8.92E-01	8.77E-01	9.66E-01	8.49E-01	7.73E-01	9.04E-01	8.74E-01
9704.0	1.13E+00	9.00E-01	8.83E-01	1.01E+00	8.74E-01	7.69E-01	9.40E-01	9.08E-01
9705.5	1.08E+00	8.91E-01	8.85E-01	1.04E+00	8.95E-01	7.85E-01	9.56E-01	9.27E-01
9707.0	1.04E+00	8.87E-01	8.73E-01	1.05E+00	8.99E-01	8.05E-01	9.81E-01	9.37E-01
9708.5	1.02E+00	9.00E-01	8.81E-01	1.06E+00	8.97E-01	8.09E-01	9.83E-01	9.49E-01
9710.0	1.06E+00	9.13E-01	8.94E-01	1.08E+00	9.07E-01	7.99E-01	9.92E-01	9.60E-01
9711.5	1.11E+00	9.42E-01	9.13E-01	1.09E+00	9.09E-01	8.03E-01	1.01E+00	9.66E-01
9712.5	1.13E+00	9.56E-01	9.28E-01	1.11E+00	9.16E-01	8.15E-01	1.02E+00	9.73E-01
9714.0	1.22E+00	9.76E-01	9.54E-01	1.13E+00	9.16E-01	8.21E-01	1.02E+00	9.79E-01
9715.5	1.26E+00	1.01E+00	9.65E-01	1.15E+00	9.12E-01	8.04E-01	1.04E+00	9.85E-01
9717.0	1.26E+00	1.04E+00	9.93E-01	1.15E+00	9.15E-01	8.43E-01	1.03E+00	9.91E-01
9718.5	1.34E+00	1.06E+00	1.01E+00	1.16E+00	9.20E-01	8.28E-01	1.04E+00	9.97E-01
9720.0	1.36E+00	1.13E+00	1.05E+00	1.19E+00	9.35E-01	8.24E-01	1.04E+00	9.98E-01

Energy (eV)	hetaerolite 34	hetaerolite 38	hydrozincite 41	junitoite	legrandite	rosasite 35	scholzite 5	smithsonite 26
9640.5	0.001993	0.002118	0.002448	0.001752	0.003376	0.001466	0.000782	-0.000254
9641.5	0.002330	-0.001139	-0.001554	0.000252	0.000719	-0.000173	-0.000995	0.000343
9643	-0.002962	0.000367	-0.000455	-0.001219	-0.001615	0.000627	0.000035	0.000062
9644.5	-0.000839	-0.000679	-0.000859	-0.001245	-0.002501	-0.002897	-0.001187	-0.000773
9646	-0.000359	-0.001622	0.000497	-0.000455	-0.001597	-0.000117	-0.000498	0.003979
9647.5	-0.003037	-0.000337	-0.001128	-0.000279	-0.000618	0.000091	-0.000344	-0.003002
9649	-0.000182	0.000832	-0.000642	-0.000583	0.000704	-0.001295	0.000653	-0.001790
9650	0.003427	0.000748	0.001749	0.002075	0.001762	0.002712	0.000709	0.001851
9651.5	-0.000196	0.004699	0.007214	0.007163	0.004417	0.005741	0.003898	0.012621
9653	0.008916	0.010952	0.013711	0.009111	0.009158	0.009433	0.006916	0.017356
9654.5	0.016527	0.026062	0.022547	0.018629	0.019836	0.015506	0.012164	0.029980
9656	0.038377	0.045913	0.033879	0.033836	0.028953	0.029258	0.028170	0.050749
9657.5	0.079115	0.103095	0.059995	0.053201	0.055446	0.056216	0.045997	0.091145
9659	0.197507	0.227531	0.112547	0.106633	0.120903	0.111601	0.086778	0.149528
9660.5	0.381425	0.421747	0.261259	0.258963	0.289820	0.253308	0.189458	0.313062
9662	0.643925	0.676354	0.676736	0.722631	0.732177	0.618308	0.428369	0.740857
9663.5	1.333152	1.357809	1.506140	1.502667	1.587117	1.386799	1.147568	1.537413
9665	2.144407	2.088614	2.228800	1.403890	2.234103	2.012003	1.612688	2.800875
9666	2.056542	1.829168	2.456378	1.335377	2.350184	2.128649	1.509012	3.993284
9667.5	1.884270	1.670474	2.664126	1.647448	2.686771	2.243233	1.358072	3.966390
9669	1.900880	1.733903	2.436375	1.909435	2.761252	2.112361	1.283327	3.117331
9670.5	1.849742	1.652395	2.075188	1.819602	2.508016	1.824312	1.253376	2.424556
9672	1.688135	1.557136	2.018843	1.650672	2.123091	1.734861	1.117878	1.851201
9673.5	1.736168	1.647610	1.795441	1.412034	1.877843	1.611107	1.040165	1.704206
9675	1.769687	1.647465	1.494730	1.225025	1.708030	1.404981	1.112595	1.671060
9676.5	1.742842	1.592039	1.315657	1.228978	1.599668	1.297410	1.199846	1.986834
9678	1.655495	1.457086	1.253080	1.224934	1.512659	1.229646	1.163471	2.281296
9679.5	1.494929	1.335797	1.259289	1.221699	1.486349	1.212944	1.116176	2.142231
9681	1.330672	1.180090	1.270123	1.204998	1.478230	1.199902	1.107336	1.943616
9682	1.232442	1.103145	1.289008	1.208383	1.432628	1.207191	1.063210	1.785764
9683.5	1.173590	1.022505	1.296145	1.218259	1.424391	1.201652	1.022004	1.501593
9685	1.168165	1.015934	1.259106	1.236199	1.364782	1.165399	0.982127	1.311514
9686.5	1.157670	1.049150	1.191646	1.192681	1.321362	1.100119	0.914134	1.226647
9688	1.141898	1.037525	1.081169	1.127949	1.261210	1.027885	0.865149	1.169471
9689.5	1.114137	0.991358	1.019650	1.078980	1.205328	0.983753	0.825739	1.111543
9691	1.082653	0.950435	0.987014	1.026665	1.200146	0.957081	0.802484	1.149226
9692.5	1.086991	0.944088	0.995827	1.004264	1.172598	0.955379	0.778059	1.197470
9694	1.111691	0.962391	1.007837	0.992912	1.171467	0.981526	0.748656	1.250753
9695.5	1.134879	0.991378	1.042042	0.980251	1.215185	1.008675	0.743531	1.358121
9696.5	1.162523	1.007931	1.046317	0.986772	1.234725	1.029843	0.758650	1.437334
9698	1.218739	1.020538	1.073827	0.979146	1.286187	1.057326	0.734233	1.507878
9699.5	1.238401	1.030156	1.106407	1.021485	1.339925	1.090392	0.757503	1.594759
9701	1.272612	1.036349	1.142806	1.061656	1.372825	1.119796	0.770960	1.734806
9702.5	1.287352	1.038433	1.211485	1.075777	1.483215	1.148699	0.809685	1.870523
9704	1.311181	1.071802	1.269301	1.102187	1.491558	1.176736	0.825092	1.951438
9705.5	1.359580	1.095646	1.309771	1.134109	1.562447	1.200752	0.846096	1.991888
9707	1.400754	1.092927	1.358946	1.171213	1.594085	1.230789	0.855487	1.955191
9708.5	1.382861	1.089265	1.394577	1.194747	1.665742	1.256958	0.850318	1.993146
9710	1.385617	1.084411	1.423400	1.211672	1.674149	1.277880	0.873935	2.023351
9711.5	1.380875	1.097465	1.425845	1.250211	1.750397	1.289533	0.889394	2.076123
9712.5	1.372978	1.098858	1.428282	1.249382	1.783315	1.289019	0.923604	2.103204
9714	1.459003	1.121429	1.404515	1.286281	1.785887	1.290108	0.923441	2.045592
9715.5	1.468961	1.158470	1.389621	1.332478	1.848409	1.289018	0.909147	2.017595
9717	1.500580	1.187089	1.406023	1.417389	1.892332	1.293932	0.929165	1.941180
9718.5	1.502447	1.198136	1.409307	1.440989	1.910337	1.290018	0.927836	1.881017
9720	1.598153	1.224461	1.416346	1.500684	1.919017	1.271864	0.948615	1.825587

Energy (eV)	sphalerite 7	sphalerite 27	tarbuttite 8	willemite 9	willemite 25	Zn acetate 10
9640.5	2.20E-04	1.03E-03	1.25E-03	1.03E-03	1.40E-03	6.38E-04
9641.5	-5.16E-04	5.44E-04	-8.64E-04	-2.13E-04	7.50E-05	5.66E-04
9643.0	5.49E-04	-1.26E-03	-1.06E-05	-9.88E-04	-9.39E-04	-2.91E-04
9644.5	-1.13E-03	-4.54E-04	-1.95E-03	-1.13E-03	-2.22E-03	-9.39E-04
9646.0	-1.39E-05	-1.59E-03	-7.05E-04	-6.04E-04	-2.31E-04	-4.07E-04
9647.5	5.67E-05	-2.22E-04	-8.41E-04	-4.10E-05	-8.30E-04	-1.01E-03
9649.0	2.46E-04	1.07E-03	1.04E-04	1.07E-03	1.45E-03	-2.41E-04
9650.0	2.71E-04	6.65E-04	1.96E-03	2.69E-04	8.42E-04	1.71E-03
9651.5	1.20E-03	5.09E-03	5.98E-03	5.58E-03	5.14E-03	4.74E-03
9653.0	1.13E-02	1.17E-02	8.51E-03	1.02E-02	8.69E-03	9.58E-03
9654.5	2.03E-02	2.12E-02	1.99E-02	1.69E-02	1.92E-02	2.27E-02
9656.0	3.56E-02	4.30E-02	3.74E-02	2.80E-02	3.15E-02	3.41E-02
9657.5	7.35E-02	7.90E-02	6.90E-02	4.91E-02	5.00E-02	6.17E-02
9659.0	1.61E-01	1.64E-01	1.12E-01	9.72E-02	9.47E-02	1.06E-01
9660.5	4.56E-01	4.69E-01	2.35E-01	2.31E-01	2.14E-01	2.49E-01
9662.0	1.04E+00	1.18E+00	5.39E-01	5.86E-01	5.52E-01	5.90E-01
9663.5	1.25E+00	1.39E+00	1.33E+00	1.23E+00	1.17E+00	1.14E+00
9665.0	1.39E+00	1.57E+00	1.95E+00	1.49E+00	1.44E+00	1.76E+00
9666.0	1.17E+00	1.35E+00	2.08E+00	1.45E+00	1.41E+00	1.89E+00
9667.5	9.70E-01	1.04E+00	2.01E+00	1.41E+00	1.38E+00	1.75E+00
9669.0	9.94E-01	1.10E+00	1.96E+00	1.34E+00	1.35E+00	1.63E+00
9670.5	9.50E-01	1.05E+00	1.80E+00	1.35E+00	1.36E+00	1.48E+00
9672.0	1.02E+00	1.09E+00	1.54E+00	1.39E+00	1.39E+00	1.38E+00
9673.5	1.05E+00	1.13E+00	1.49E+00	1.32E+00	1.30E+00	1.33E+00
9675.0	1.02E+00	1.12E+00	1.49E+00	1.24E+00	1.22E+00	1.30E+00
9676.5	1.04E+00	1.09E+00	1.41E+00	1.18E+00	1.18E+00	1.28E+00
9678.0	1.02E+00	1.08E+00	1.37E+00	1.12E+00	1.12E+00	1.30E+00
9679.5	9.57E-01	1.02E+00	1.36E+00	1.10E+00	1.09E+00	1.29E+00
9681.0	9.50E-01	9.83E-01	1.31E+00	1.12E+00	1.13E+00	1.27E+00
9682.0	9.16E-01	9.68E-01	1.27E+00	1.13E+00	1.14E+00	1.22E+00
9683.5	9.34E-01	9.76E-01	1.22E+00	1.10E+00	1.11E+00	1.17E+00
9685.0	9.38E-01	1.00E+00	1.19E+00	1.05E+00	1.07E+00	1.13E+00
9686.5	9.73E-01	1.02E+00	1.14E+00	1.01E+00	9.96E-01	1.05E+00
9688.0	9.92E-01	1.04E+00	1.10E+00	9.61E-01	9.66E-01	1.02E+00
9689.5	9.94E-01	1.03E+00	1.05E+00	9.13E-01	9.11E-01	9.72E-01
9691.0	9.36E-01	1.00E+00	1.02E+00	8.85E-01	8.91E-01	9.49E-01
9692.5	9.04E-01	9.60E-01	9.90E-01	8.66E-01	8.85E-01	9.31E-01
9694.0	8.75E-01	9.21E-01	1.03E+00	8.61E-01	8.84E-01	9.25E-01
9695.5	8.48E-01	8.98E-01	1.06E+00	8.61E-01	8.92E-01	9.30E-01
9696.5	8.29E-01	8.99E-01	1.09E+00	8.65E-01	8.95E-01	9.55E-01
9698.0	8.28E-01	8.85E-01	1.12E+00	8.60E-01	8.80E-01	9.81E-01
9699.5	8.13E-01	8.73E-01	1.17E+00	8.67E-01	8.85E-01	1.01E+00
9701.0	8.34E-01	8.74E-01	1.18E+00	8.72E-01	8.82E-01	1.03E+00
9702.5	8.20E-01	8.78E-01	1.24E+00	8.77E-01	8.92E-01	1.04E+00
9704.0	8.21E-01	9.13E-01	1.27E+00	8.87E-01	9.09E-01	1.08E+00
9705.5	8.31E-01	9.23E-01	1.32E+00	9.03E-01	9.31E-01	1.11E+00
9707.0	8.73E-01	9.57E-01	1.37E+00	9.24E-01	9.51E-01	1.16E+00
9708.5	8.91E-01	9.80E-01	1.37E+00	9.44E-01	9.57E-01	1.17E+00
9710.0	9.02E-01	9.95E-01	1.38E+00	9.75E-01	9.93E-01	1.18E+00
9711.5	9.11E-01	9.94E-01	1.42E+00	1.01E+00	1.02E+00	1.21E+00
9712.5	8.76E-01	9.88E-01	1.43E+00	1.02E+00	1.03E+00	1.21E+00
9714.0	8.88E-01	9.95E-01	1.43E+00	1.04E+00	1.06E+00	1.23E+00
9715.5	8.87E-01	1.01E+00	1.47E+00	1.06E+00	1.07E+00	1.23E+00
9717.0	8.81E-01	1.02E+00	1.46E+00	1.08E+00	1.10E+00	1.24E+00
9718.5	8.95E-01	1.02E+00	1.50E+00	1.09E+00	1.11E+00	1.25E+00
9720.0	9.13E-01	1.04E+00	1.51E+00	1.08E+00	1.10E+00	1.25E+00

Energy (eV)	Zn bis()imide 20	Zn bromide	Zn chloride	Zn citrate	Zn carbonate	Zn nitrate
9640.5	2.03E-03	-1.07E-04	9.23E-04	1.67E-03	1.01E-03	2.91E-03
9641.5	9.48E-04	6.64E-04	-9.28E-04	4.00E-04	1.11E-03	1.07E-03
9643.0	-4.75E-04	-1.33E-03	1.60E-04	-6.57E-04	2.73E-04	-2.67E-03
9644.5	-3.79E-03	-1.47E-04	-7.39E-04	-1.44E-03	-2.70E-03	-3.96E-03
9646.0	-2.46E-04	4.00E-04	-9.53E-04	-3.14E-03	-5.29E-04	-2.04E-03
9647.5	-3.28E-03	7.91E-04	-2.36E-03	-8.35E-04	-1.45E-03	-8.97E-04
9649.0	2.18E-04	4.66E-04	2.05E-03	7.24E-04	-6.10E-04	1.89E-03
9650.0	3.94E-03	1.08E-05	8.52E-04	2.65E-03	2.95E-03	2.87E-03
9651.5	1.80E-02	4.23E-03	5.04E-03	3.96E-03	5.35E-03	1.10E-02
9653.0	2.88E-02	1.27E-02	7.67E-03	9.04E-03	1.13E-02	1.69E-02
9654.5	6.20E-02	2.36E-02	1.56E-02	1.88E-02	2.59E-02	4.06E-02
9656.0	1.10E-01	3.61E-02	1.96E-02	2.99E-02	3.93E-02	7.35E-02
9657.5	1.60E-01	6.60E-02	3.86E-02	5.37E-02	8.62E-02	1.45E-01
9659.0	2.32E-01	1.14E-01	7.22E-02	1.02E-01	1.33E-01	2.11E-01
9660.5	3.46E-01	2.62E-01	1.91E-01	2.22E-01	2.75E-01	3.55E-01
9662.0	5.76E-01	6.41E-01	6.14E-01	5.45E-01	6.41E-01	6.28E-01
9663.5	1.23E+00	1.66E+00	2.74E+00	1.12E+00	1.33E+00	1.24E+00
9665.0	1.80E+00	1.86E+00	2.99E+00	2.03E+00	1.89E+00	1.76E+00
9666.0	1.94E+00	1.71E+00	2.45E+00	2.39E+00	2.10E+00	1.84E+00
9667.5	2.04E+00	1.54E+00	2.13E+00	2.50E+00	2.28E+00	1.89E+00
9669.0	1.94E+00	1.44E+00	1.91E+00	2.24E+00	2.06E+00	1.84E+00
9670.5	1.75E+00	1.36E+00	1.72E+00	1.84E+00	1.80E+00	1.66E+00
9672.0	1.56E+00	1.29E+00	1.60E+00	1.53E+00	1.71E+00	1.46E+00
9673.5	1.39E+00	1.25E+00	1.46E+00	1.39E+00	1.54E+00	1.28E+00
9675.0	1.28E+00	1.20E+00	1.36E+00	1.32E+00	1.33E+00	1.15E+00
9676.5	1.19E+00	1.16E+00	1.34E+00	1.33E+00	1.19E+00	1.09E+00
9678.0	1.16E+00	1.13E+00	1.26E+00	1.34E+00	1.14E+00	1.12E+00
9679.5	1.13E+00	1.11E+00	1.25E+00	1.34E+00	1.16E+00	1.12E+00
9681.0	1.11E+00	1.08E+00	1.25E+00	1.33E+00	1.19E+00	1.10E+00
9682.0	1.12E+00	1.06E+00	1.25E+00	1.30E+00	1.20E+00	1.09E+00
9683.5	1.07E+00	1.05E+00	1.21E+00	1.24E+00	1.20E+00	1.10E+00
9685.0	1.07E+00	1.05E+00	1.19E+00	1.18E+00	1.14E+00	1.05E+00
9686.5	1.03E+00	1.03E+00	1.20E+00	1.14E+00	1.09E+00	9.61E-01
9688.0	9.83E-01	1.01E+00	1.20E+00	1.14E+00	1.03E+00	9.35E-01
9689.5	9.32E-01	1.03E+00	1.17E+00	1.13E+00	9.58E-01	9.09E-01
9691.0	9.25E-01	1.01E+00	1.17E+00	1.14E+00	9.22E-01	8.78E-01
9692.5	9.16E-01	1.02E+00	1.17E+00	1.11E+00	9.49E-01	8.61E-01
9694.0	8.93E-01	1.02E+00	1.14E+00	1.10E+00	9.39E-01	8.67E-01
9695.5	9.08E-01	1.02E+00	1.13E+00	1.12E+00	9.59E-01	8.88E-01
9696.5	9.01E-01	1.03E+00	1.08E+00	1.13E+00	9.90E-01	9.06E-01
9698.0	9.16E-01	1.04E+00	1.13E+00	1.17E+00	9.67E-01	9.07E-01
9699.5	9.52E-01	1.05E+00	1.10E+00	1.19E+00	1.00E+00	9.28E-01
9701.0	9.83E-01	1.06E+00	1.17E+00	1.24E+00	1.05E+00	9.73E-01
9702.5	1.01E+00	1.08E+00	1.16E+00	1.28E+00	1.07E+00	9.88E-01
9704.0	1.03E+00	1.09E+00	1.15E+00	1.30E+00	1.11E+00	1.01E+00
9705.5	1.05E+00	1.09E+00	1.20E+00	1.34E+00	1.16E+00	1.03E+00
9707.0	1.09E+00	1.11E+00	1.20E+00	1.39E+00	1.18E+00	1.04E+00
9708.5	1.11E+00	1.12E+00	1.27E+00	1.41E+00	1.23E+00	1.04E+00
9710.0	1.12E+00	1.10E+00	1.31E+00	1.43E+00	1.24E+00	1.05E+00
9711.5	1.11E+00	1.11E+00	1.35E+00	1.46E+00	1.23E+00	1.07E+00
9712.5	1.10E+00	1.12E+00	1.39E+00	1.46E+00	1.21E+00	1.05E+00
9714.0	1.11E+00	1.12E+00	1.44E+00	1.49E+00	1.21E+00	1.04E+00
9715.5	1.09E+00	1.11E+00	1.49E+00	1.50E+00	1.20E+00	1.03E+00
9717.0	1.09E+00	1.11E+00	1.52E+00	1.50E+00	1.20E+00	1.03E+00
9718.5	1.07E+00	1.10E+00	1.58E+00	1.51E+00	1.20E+00	1.03E+00
9720.0	1.05E+00	1.11E+00	1.63E+00	1.50E+00	1.20E+00	9.80E-01

Energy (eV)	Zn oxide	Zn phosphate 17	Zn protoporphyrin	Zn stearate 18	Zn sulfate 19	Zn sulfide 23
9640.5	1.52E-03	5.73E-04	1.18E-03	1.27E-03	1.40E-03	1.20E-03
9641.5	1.41E-04	-2.52E-04	7.59E-04	2.34E-04	1.48E-03	2.89E-04
9643.0	-1.23E-03	5.61E-04	-1.19E-03	-1.06E-03	-1.94E-03	-1.34E-03
9644.5	-1.21E-03	1.30E-04	-1.81E-03	-5.38E-04	-1.05E-03	-1.32E-03
9646.0	-1.38E-03	-2.97E-03	3.56E-04	-2.91E-03	-9.47E-04	-4.21E-04
9647.5	-8.78E-04	-2.92E-04	-1.53E-03	1.72E-03	-2.39E-04	-4.42E-04
9649.0	3.32E-04	4.78E-04	2.27E-04	-2.47E-03	-4.89E-04	3.85E-04
9650.0	2.05E-03	1.36E-03	1.91E-03	3.26E-03	2.25E-03	1.19E-03
9651.5	3.45E-03	6.22E-03	4.09E-03	8.44E-03	2.69E-03	7.55E-03
9653.0	8.03E-03	1.02E-02	1.16E-02	2.01E-02	6.53E-03	1.36E-02
9654.5	1.91E-02	2.04E-02	2.97E-02	3.11E-02	1.41E-02	2.36E-02
9656.0	2.85E-02	3.42E-02	5.23E-02	6.49E-02	2.68E-02	4.17E-02
9657.5	5.90E-02	5.55E-02	1.11E-01	1.05E-01	4.48E-02	8.10E-02
9659.0	1.27E-01	9.33E-02	2.44E-01	1.55E-01	8.32E-02	1.54E-01
9660.5	3.33E-01	2.05E-01	7.32E-01	2.86E-01	1.83E-01	4.57E-01
9662.0	7.83E-01	5.01E-01	9.44E-01	6.01E-01	3.97E-01	1.12E+00
9663.5	1.08E+00	1.25E+00	6.56E-01	1.08E+00	9.84E-01	1.34E+00
9665.0	1.13E+00	1.91E+00	9.13E-01	1.69E+00	1.75E+00	1.50E+00
9666.0	1.25E+00	1.92E+00	1.23E+00	1.73E+00	1.85E+00	1.29E+00
9667.5	1.56E+00	1.80E+00	1.43E+00	1.55E+00	1.70E+00	1.03E+00
9669.0	1.79E+00	1.72E+00	1.56E+00	1.48E+00	1.64E+00	1.08E+00
9670.5	1.61E+00	1.56E+00	1.66E+00	1.32E+00	1.52E+00	1.04E+00
9672.0	1.30E+00	1.36E+00	1.62E+00	1.20E+00	1.35E+00	1.09E+00
9673.5	1.11E+00	1.24E+00	1.46E+00	1.16E+00	1.21E+00	1.16E+00
9675.0	9.42E-01	1.25E+00	1.35E+00	1.19E+00	1.12E+00	1.15E+00
9676.5	9.27E-01	1.28E+00	1.24E+00	1.17E+00	1.08E+00	1.11E+00
9678.0	1.06E+00	1.25E+00	1.13E+00	1.21E+00	1.04E+00	1.11E+00
9679.5	1.16E+00	1.19E+00	1.08E+00	1.22E+00	1.03E+00	1.06E+00
9681.0	1.12E+00	1.13E+00	1.06E+00	1.22E+00	9.86E-01	1.03E+00
9682.0	1.08E+00	1.11E+00	1.04E+00	1.17E+00	9.69E-01	1.02E+00
9683.5	1.04E+00	1.11E+00	9.90E-01	1.11E+00	9.37E-01	1.05E+00
9685.0	9.71E-01	1.06E+00	9.29E-01	1.00E+00	9.00E-01	1.05E+00
9686.5	9.23E-01	1.00E+00	8.89E-01	9.10E-01	8.54E-01	1.08E+00
9688.0	8.85E-01	9.66E-01	8.49E-01	8.77E-01	8.14E-01	1.10E+00
9689.5	8.51E-01	9.04E-01	8.46E-01	8.38E-01	7.76E-01	1.09E+00
9691.0	7.95E-01	8.75E-01	8.56E-01	8.04E-01	7.50E-01	1.08E+00
9692.5	7.40E-01	8.33E-01	8.55E-01	7.79E-01	7.31E-01	1.02E+00
9694.0	7.26E-01	8.31E-01	8.90E-01	7.65E-01	7.27E-01	9.95E-01
9695.5	7.09E-01	8.41E-01	8.89E-01	7.62E-01	7.12E-01	9.65E-01
9696.5	7.01E-01	8.40E-01	8.81E-01	7.83E-01	7.01E-01	9.88E-01
9698.0	6.98E-01	8.56E-01	8.83E-01	7.75E-01	7.16E-01	9.87E-01
9699.5	7.08E-01	8.79E-01	8.67E-01	7.91E-01	7.18E-01	9.76E-01
9701.0	7.32E-01	9.07E-01	8.80E-01	8.20E-01	7.46E-01	9.69E-01
9702.5	7.58E-01	9.29E-01	9.21E-01	8.65E-01	7.58E-01	9.78E-01
9704.0	7.80E-01	9.65E-01	9.69E-01	8.67E-01	7.81E-01	1.00E+00
9705.5	8.10E-01	1.02E+00	1.03E+00	8.90E-01	7.87E-01	1.03E+00
9707.0	8.26E-01	1.04E+00	1.08E+00	9.31E-01	7.76E-01	1.06E+00
9708.5	8.46E-01	1.04E+00	1.13E+00	9.34E-01	7.75E-01	1.08E+00
9710.0	8.79E-01	1.06E+00	1.13E+00	9.72E-01	7.67E-01	1.11E+00
9711.5	9.09E-01	1.06E+00	1.12E+00	9.75E-01	7.51E-01	1.11E+00
9712.5	9.42E-01	1.09E+00	1.11E+00	9.90E-01	7.47E-01	1.12E+00
9714.0	9.78E-01	1.11E+00	1.10E+00	9.83E-01	7.16E-01	1.12E+00
9715.5	9.63E-01	1.11E+00	1.07E+00	9.86E-01	6.83E-01	1.12E+00
9717.0	9.14E-01	1.09E+00	1.07E+00	9.71E-01	6.78E-01	1.15E+00
9718.5	8.57E-01	1.10E+00	1.06E+00	9.62E-01	6.42E-01	1.17E+00
9720.0	8.19E-01	1.09E+00	1.03E+00	9.45E-01	6.16E-01	1.19E+00

Table S2 Zinc Mineral X-ray Diffraction (XRD)

**descloizite 29**		Pos. [°2θ]	Height [cts]	FWHM Left [°2θ]	d-spacing [Å]	Rel. Int. [%]
Scan Axis	Gonio	17.595	19.13	0.1023	5.04068	36.96
Start Position [°2θ]	15.0091	19.0759	12.08	0.1023	4.65259	23.34
End Position [°2θ]	84.9881	21.2161	11.71	0.1023	4.18785	22.63
Step Size [°2θ]	0.013	22.4156	8.2	0.1535	3.96637	15.85
Scan Step Time [s]	8.67	25.4963	12.64	0.1279	3.49369	24.42
Scan Type	Continuous	26.9143	2.87	0.1279	3.31275	5.54
PSD Mode	Scanning	27.9189	51.75	0.0384	3.19579	100
PSD Length [°2θ]	2	29.6592	3.85	0.1535	3.01212	7.44
Offset [°2θ]	0	30.4741	1.9	0.1535	2.9334	3.68
Divergence Slit Type	Fixed	31.0233	21.85	0.1535	2.88271	42.21
Divergence Slit Size [°]	0.0573	33.4023	12.85	0.1535	2.68264	24.82
Specimen Length [mm]	10	33.9455	7.66	0.1791	2.64095	14.81
Measurement Temperature [°C]	25	34.4533	25.67	0.064	2.60318	49.6
Anode Material	Cu	35.4269	2.69	0.1535	2.53383	5.2
K-Alpha1 [Å]	1.5406	36.9937	5.52	0.1535	2.43004	10.66
K-Alpha2 [Å]	1.54443	39.4283	5.89	0.1535	2.28543	11.38
K-Beta [Å]	1.39225	40.2998	5.12	0.1791	2.23799	9.9
K-A2 / K-A1 Ratio	0.5	40.6411	2.45	0.1535	2.21998	4.74
Generator Settings	40 mA, 45	43.4054	4.97	0.1535	2.0848	9.6
Diffractometer Type	11171041	46.1694	1.59	0.307	1.96622	3.07
Diffractometer Number	0	48.0882	3.57	0.1535	1.89215	6.9
Goniometer Radius [mm]	240	51.4291	2.71	0.2047	1.77681	5.24
Dist. Focus-Diverg. Slit [mm]	100	52.1295	4.48	0.1535	1.75458	8.65
Incident Beam Monochromator	No	53.2969	4.47	0.1535	1.71886	8.64
Spinning	Yes	55.8249	5.64	0.307	1.64687	10.91
		57.5465	0.63	0.614	1.60163	1.22
		59.1823	2.52	0.307	1.56121	4.88
		61.6063	1.06	0.614	1.50547	2.05
		63.4847	1	0.8187	1.46538	1.93
		66.4111	4.36	0.2047	1.40773	8.43
		75.0639	2.04	0.307	1.26548	3.94
		77.2876	4.65	0.1248	1.23351	8.98

**franklinite 22**		Pos. [°2θ]	Height [cts]	FWHM Left [°2θ]	d-spacing [Å]	Rel. Int. [%]
Scan Axis	Gonio					
Start Position [°2θ]	10.0066	35.2048	58.77	0.0468	2.5472	56.4
End Position [°2θ]	79.9856	36.3064	17.6	0.1535	2.47445	16.89
Step Size [°2θ]	0.013	70.1856	104.2	0.0468	1.33988	100
Scan Step Time [s]	37.995					
Scan Type	Continuous					
PSD Mode	Scanning					
PSD Length [°2θ]	2					
Offset [°2θ]	0					
Divergence Slit Type	Fixed					
Divergence Slit Size [°]	0.0573					
Specimen Length [mm]	10					
Measurement Temperature [°C]	25					
Anode Material	Cu					
K-Alpha1 [Å]	1.5406					
K-Alpha2 [Å]	1.54443					
K-Beta [Å]	1.39225					
K-A2 / K-A1 Ratio	0.5					
Generator Settings	40 mA, 45 kV					
Diffractometer Type	11171041					
Diffractometer Number	0					
Goniometer Radius [mm]	240					
Dist. Focus-Diverg. Slit [mm]	100					
Incident Beam Monochromator	No					
Spinning	Yes					

**gahnite 33**		Pos. [°2θ]	Height [cts]	FWHM Left [°2θ]	d-spacing [Å]	Rel. Int. [%]
Scan Axis	Gonio	31.4699	114.34	0.0624	2.84047	58.83
Start Position [°2θ]	10.0091	31.5473	68.97	0.0468	2.84071	35.49
End Position [°2θ]	79.9881	37.0284	194.35	0.0468	2.42583	100
Step Size [°2θ]	0.013	37.123	94.82	0.0468	2.42589	48.79
Scan Step Time [s]	8.67	45.0954	30.15	0.0468	2.00885	15.51
Scan Type	Continuous	49.2737	2.75	0.3744	1.84783	1.41
PSD Mode	Scanning	55.7806	17.96	0.078	1.64671	9.24
PSD Length [°2θ]	2	55.9583	14.52	0.0936	1.6419	7.47
Offset [°2θ]	0	59.5044	140.82	0.0624	1.55224	72.46
Divergence Slit Type	Fixed	59.6361	60.49	0.0624	1.55298	31.12
Divergence Slit Size [°]	0.0573	65.3493	35.83	0.0624	1.42683	18.44
Specimen Length [mm]	10	74.2392	4.6	0.3744	1.27643	2.37
Measurement Temperature [°C]	25	77.433	35.78	0.0468	1.23156	18.41
Anode Material	Cu					
K-Alpha1 [Å]	1.5406					
K-Alpha2 [Å]	1.54443					
K-Beta [Å]	1.39225					
K-A2 / K-A1 Ratio	0.5					
Generator Settings	40 mA, 45 kV					
Diffractometer Type	11171041					
Diffractometer Number	0					
Goniometer Radius [mm]	240					
Dist. Focus-Diverg. Slit [mm]	100					
Incident Beam Monochromator	No					
Spinning	Yes					

**hardystonite 32**		Pos. [°2θ]	Height [cts]	FWHM Left [°2θ]	d-spacing [Å]	Rel. Int. [%]
Scan Axis	Gonio	17.8432	23.92	0.1023	4.97113	13.06
Start Position [°2θ]	10.0066	21.2359	20.46	0.1023	4.18397	11.18
End Position [°2θ]	79.9856	24.1317	61.25	0.1279	3.68806	33.46
Step Size [°2θ]	0.013	25.5779	3.6	0.2558	3.48272	1.97
Scan Step Time [s]	8.67	29.1343	66.01	0.1023	3.06518	36.06
Scan Type	Continuous	31.3571	183.06	0.1279	2.85278	100
PSD Mode	Scanning	32.5359	8.25	0.307	2.75207	4.51
PSD Length [°2θ]	2	33.4766	5.75	0.1535	2.67686	3.14
Offset [°2θ]	0	35.3927	15.59	0.1023	2.53621	8.52
Divergence Slit Type	Fixed	35.9936	8.04	0.1535	2.49523	4.39
Divergence Slit Size [°]	0.0573	36.4813	29.17	0.1023	2.46299	15.94
Specimen Length [mm]	10	37.2519	8.95	0.2558	2.41379	4.89
Measurement Temperature [°C]	25	37.8535	9.1	0.1535	2.3768	4.97
Anode Material	Cu	39.1156	5.43	0.3582	2.30297	2.97
K-Alpha1 [Å]	1.5406	39.6468	12.03	0.1535	2.27333	6.57
K-Alpha2 [Å]	1.54443	40.8758	7.23	0.2047	2.20778	3.95
K-Beta [Å]	1.39225	42.9533	9.03	0.09	2.10394	4.93
K-A2 / K-A1 Ratio	0.5	44.6157	17.63	0.2047	2.03101	9.63
Generator Settings	40 mA, 45	45.7371	3.46	0.2558	1.98379	1.89
Diffractometer Type	11171041	46.6701	3.76	0.2047	1.94628	2.05
Diffractometer Number	0	48.1086	6.92	0.2047	1.89139	3.78
Goniometer Radius [mm]	240	49.1582	16.7	0.1279	1.85344	9.12
Dist. Focus-Diverg. Slit [mm]	100	49.6473	6.79	0.1535	1.83632	3.71
Incident Beam Monochromator	No	52.0807	36.44	0.2047	1.7561	19.91
Spinning	No	53.0907	10.33	0.2558	1.72505	5.64
		55.8326	5.54	0.307	1.64666	3.03
		57.7206	10.7	0.2558	1.59722	5.85
		60.3539	9.46	0.2047	1.53368	5.17
		61.5899	8.59	0.307	1.50583	4.69
		63.5299	6.26	0.1535	1.46445	3.42
		65.2442	8.82	0.4093	1.43006	4.82
		66.5745	4.96	0.2047	1.40467	2.71
		67.1902	10.58	0.2047	1.39329	5.78
		67.8378	5.38	0.307	1.38156	2.94
		70.8133	3.25	0.8187	1.33063	1.78
		72.6491	7.28	0.2047	1.30147	3.97
		75.9424	8.54	0.2558	1.25302	4.67
		77.1111	4.56	0.4093	1.23692	2.49

**hardystonite 40**		Pos. [°2θ]	Height [cts]	FWHM Left [°2θ]	d-spacing [Å]	Rel. Int. [%]
Scan Axis	Gonio	17.9031	34.22	0.0512	4.95463	98.28
Start Position [°2θ]	10.0091	24.1801	18.13	0.1279	3.68079	52.06
End Position [°2θ]	79.9881	29.1514	28.79	0.0895	3.06342	82.69
Step Size [°2θ]	0.013	31.3763	34.82	0.1535	2.85108	100
Scan Step Time [s]	8.67	32.637	1.12	0.307	2.74378	3.2
Scan Type	Continuous	35.9923	13.32	0.0468	2.49326	38.25
PSD Mode	Scanning	36.5373	12.82	0.2047	2.45934	36.82
PSD Length [°2θ]	2	40.8315	1.14	0.614	2.21007	3.28
Offset [°2θ]	0	44.6739	2.51	0.2047	2.0285	7.22
Divergence Slit Type	Fixed	48.1757	1.65	0.4093	1.88892	4.75
Divergence Slit Size [°]	0.0573	52.1201	10.09	0.1535	1.75487	28.98
Specimen Length [mm]	10	61.6171	2.46	0.1535	1.50523	7.05
Measurement Temperature [°C]	25	63.5994	1.91	0.307	1.46301	5.49
Anode Material	Cu	65.3549	1.32	0.307	1.4279	3.79
K-Alpha1 [Å]	1.5406	76.041	1	0.614	1.25164	2.86
K-Alpha2 [Å]	1.54443					
K-Beta [Å]	1.39225					
K-A2 / K-A1 Ratio	0.5					
Generator Settings	40 mA, 45 kV					
Diffractometer Type	11171041					
Diffractometer Number	0					
Goniometer Radius [mm]	240					
Dist. Focus-Diverg. Slit [mm]	100					
Incident Beam Monochromator	No					
Spinning	Yes					

**hemimorphite 4**		Pos. [°2θ]	Height [cts]	FWHM Left [°2θ]	d-spacing [Å]	Rel. Int. [%]
Scan Axis	Gonio	13.4197	360.96	0.0768	6.59816	100
Start Position [°2θ]	10.0066	16.5364	226.09	0.0512	5.36091	62.64
End Position [°2θ]	79.9856	19.2194	46.25	0.0512	4.61817	12.81
Step Size [°2θ]	0.013	21.2345	29.84	0.0768	4.18425	8.27
Scan Step Time [s]	18.87	27.0177	115.85	0.0468	3.29758	32.1
Scan Type	Continuous	27.1286	169.41	0.064	3.28707	46.93
PSD Mode	Scanning	28.7802	155.53	0.0468	3.09951	43.09
PSD Length [°2θ]	2	28.8629	76.94	0.0468	3.0985	21.32
Offset [°2θ]	0	30.5111	47.68	0.0468	2.92751	13.21
Divergence Slit Type	Fixed	33.4202	18.01	0.156	2.67903	4.99
Divergence Slit Size [°]	0.0573	35.0617	24.05	0.0624	2.55727	6.66
Specimen Length [mm]	10	36.6689	19.4	0.0624	2.44879	5.38
Measurement Temperature [°C]	25	37.4594	20.19	0.0468	2.39891	5.59
Anode Material	Cu	40.5297	4.63	0.1872	2.22398	1.28
K-Alpha1 [Å]	1.5406	41.0186	56.16	0.0624	2.1986	15.56
K-Alpha2 [Å]	1.54443	41.1337	33.9	0.0468	2.19816	9.39
K-Beta [Å]	1.39225	43.214	0.98	0.8736	2.09185	0.27
K-A2 / K-A1 Ratio	0.5	44.8723	5.16	0.1872	2.01832	1.43
Generator Settings	40 mA, 45 kV	50.501	2.89	0.1872	1.80577	0.8
Diffractometer Type	11171041	51.1063	32.59	0.0624	1.7858	9.03
Diffractometer Number	0	53.7842	13.22	0.0624	1.70303	3.66
Goniometer Radius [mm]	240	55.5405	33.3	0.078	1.65326	9.23
Dist. Focus-Diverg. Slit [mm]	100	58.0029	3.11	0.312	1.5888	0.86
Incident Beam Monochromator	No	59.1008	2.21	0.3744	1.56187	0.61
Spinning	No	60.6872	14.39	0.0936	1.52479	3.99
		61.0955	21.19	0.0936	1.51557	5.87
		63.498	25.28	0.078	1.46389	7
		64.4715	9.04	0.312	1.44412	2.5
		69.0895	11.8	0.0936	1.35843	3.27
		72.5397	1.98	0.3744	1.30208	0.55

**hemimorphite 6**		Pos. [°2θ]	Height [cts]	FWHM Left [°2θ]	d-spacing [Å]	Rel. Int. [%]
Scan Axis	Gonio	13.5222	85.51	0.064	6.54837	60.88
Start Position [°2θ]	10.0038	16.5944	27.29	0.1535	5.34232	19.43
End Position [°2θ]	89.9928	19.288	23.54	0.1535	4.6019	16.76
Step Size [°2θ]	0.013	21.3835	44.45	0.1023	4.15544	31.65
Scan Step Time [s]	18.87	26.3031	6.5	0.1535	3.38833	4.62
Scan Type	Continuous	27.225	54.28	0.1791	3.27565	38.65
PSD Mode	Scanning	28.8401	140.46	0.0512	3.09577	100
PSD Length [°2θ]	2	29.517	8.29	0.1535	3.0263	5.91
Offset [°2θ]	0	30.5686	30.47	0.1023	2.92455	21.69
Divergence Slit Type	Fixed	33.3404	2.56	0.614	2.68748	1.82
Divergence Slit Size [°]	0.0573	35.0923	15.63	0.1023	2.55723	11.13
Specimen Length [mm]	10	36.7053	20.76	0.1023	2.44847	14.78
Measurement Temperature [°C]	25	37.4978	35.66	0.1279	2.39853	25.39
Anode Material	Cu	40.5123	8.4	0.1791	2.22674	5.98
K-Alpha1 [Å]	1.5406	41.094	18.21	0.1279	2.19655	12.96
K-Alpha2 [Å]	1.54443	41.4187	14.15	0.1535	2.18008	10.07
K-Beta [Å]	1.39225	43.3723	7.01	0.4093	2.08631	4.99
K-A2 / K-A1 Ratio	0.5	44.9355	9.75	0.2047	2.0173	6.94
Generator Settings	40 mA, 45	45.9244	5.66	0.1535	1.97613	4.03
Diffractometer Type	11171041	49.2127	2.71	0.307	1.85152	1.93
Diffractometer Number	0	50.5041	7.45	0.2047	1.80716	5.3
Goniometer Radius [mm]	240	51.1516	9.06	0.2047	1.7858	6.45
Dist. Focus-Diverg. Slit [mm]	100	54.1741	14.04	0.1023	1.69309	9.99
Incident Beam Monochromator	No	55.1226	4.76	0.2558	1.66618	3.39
Spinning	Yes	55.7751	3.09	0.4093	1.64822	2.2
		56.8796	1.99	0.307	1.61882	1.42
		59.2979	6.7	0.307	1.55844	4.77
		60.1714	3.75	0.307	1.53789	2.67
		61.156	7.39	0.1535	1.51547	5.26
		63.5016	10.44	0.1279	1.46503	7.43
		64.0061	5.57	0.1535	1.4547	3.96
		64.5326	19.64	0.1279	1.44409	13.98
		67.7397	7.52	0.4093	1.38332	5.35
		72.5607	3.63	0.4093	1.30284	2.58
		86.2866	2.44	0.307	1.12739	1.74
		87.1109	5.14	0.0936	1.1179	3.66

**hemimorphite 36**		Pos. [°2θ]	Height [cts]	FWHM Left [°2θ]	d-spacing [Å]	Rel. Int. [%]
Scan Axis	Gonio	13.8016	91.27	0.0768	6.41643	80.2
Start Position [°2θ]	10.0091	16.927	81.88	0.064	5.23808	71.95
End Position [°2θ]	79.9881	19.6066	21.33	0.1023	4.52784	18.75
Step Size [°2θ]	0.013	21.6228	40.35	0.0895	4.10998	35.46
Scan Step Time [s]	8.67	27.067	4.14	0.1535	3.2944	3.63
Scan Type	Continuous	27.5082	88.31	0.0512	3.24257	77.61
PSD Mode	Scanning	29.1654	113.79	0.0512	3.06198	100
PSD Length [°2θ]	2	30.8892	46.26	0.0512	2.89493	40.65
Offset [°2θ]	0	33.8472	5.66	0.1535	2.64839	4.97
Divergence Slit Type	Fixed	35.4547	38.16	0.1279	2.53191	33.54
Divergence Slit Size [°]	0.0573	37.0693	19.44	0.1023	2.42526	17.08
Specimen Length [mm]	10	37.8327	64.04	0.064	2.37806	56.28
Measurement Temperature [°C]	25	40.8888	6.68	0.2047	2.2071	5.87
Anode Material	Cu	41.4003	22.8	0.1023	2.18101	20.04
K-Alpha1 [Å]	1.5406	43.6071	7.11	0.2047	2.07562	6.25
K-Alpha2 [Å]	1.54443	45.2626	8.67	0.2047	2.00348	7.62
K-Beta [Å]	1.39225	49.6304	3.11	0.4093	1.83691	2.74
K-A2 / K-A1 Ratio	0.5	50.8194	16.39	0.1023	1.79669	14.4
Generator Settings	40 mA, 45	51.5247	14.9	0.2558	1.77374	13.09
Diffractometer Type	11171041	55.4097	10.06	0.1023	1.65822	8.84
Diffractometer Number	0	56.0722	4.37	0.4093	1.64019	3.84
Goniometer Radius [mm]	240	57.1689	4.34	0.0768	1.61131	3.81
Dist. Focus-Diverg. Slit [mm]	100	58.3136	1.99	0.307	1.58238	1.75
Incident Beam Monochromator	No	61.453	13.6	0.1535	1.50886	11.95
Spinning	Yes	63.8446	8.52	0.307	1.45799	7.49
		64.8619	13.28	0.2047	1.43756	11.67
		68.0462	7.07	0.4093	1.37784	6.21
		68.8794	5.8	0.1023	1.36319	5.1
		69.4848	1.97	0.307	1.35279	1.73
		72.8503	4.45	0.4093	1.29837	3.91
		74.5183	2.19	0.4093	1.27339	1.93

**hemimorphite 37**		Pos. [°2θ]	Height [cts]	FWHM Left [°2θ]	d-spacing [Å]	Rel. Int. [%]
Scan Axis	Gonio	13.6934	78.94	0.0895	6.46686	55.07
Start Position [°2θ]	10.0091	16.836	88.85	0.1023	5.26619	61.99
End Position [°2θ]	79.9881	19.496	23.92	0.1023	4.55327	16.69
Step Size [°2θ]	0.013	21.1823	7.67	0.1535	4.19445	5.35
Scan Step Time [s]	8.67	21.5157	41.53	0.1023	4.1302	28.97
Scan Type	Continuous	26.9428	19.71	0.1023	3.30931	13.75
PSD Mode	Scanning	27.4054	143.34	0.0512	3.25449	100
PSD Length [°2θ]	2	29.0495	93.83	0.064	3.07393	65.46
Offset [°2θ]	0	30.7638	56.85	0.0512	2.90644	39.66
Divergence Slit Type	Fixed	33.7082	16.27	0.1279	2.65899	11.35
Divergence Slit Size [°]	0.0573	35.3243	23.15	0.1279	2.54096	16.15
Specimen Length [mm]	10	36.9284	17.6	0.1279	2.43419	12.28
Measurement Temperature [°C]	25	37.7104	43.51	0.0512	2.38549	30.36
Anode Material	Cu	40.7464	6.28	0.1535	2.21449	4.38
K-Alpha1 [Å]	1.5406	41.2984	14.77	0.1279	2.18616	10.3
K-Alpha2 [Å]	1.54443	43.498	3.2	0.1535	2.08057	2.24
K-Beta [Å]	1.39225	45.1486	6.84	0.2047	2.00827	4.77
K-A2 / K-A1 Ratio	0.5	49.4477	3.64	0.307	1.84326	2.54
Generator Settings	40 mA, 45	50.683	14.59	0.0768	1.8012	10.18
Diffractometer Type	11171041	51.3548	25.53	0.0768	1.77921	17.81
Diffractometer Number	0	54.3052	1.68	0.614	1.68931	1.17
Goniometer Radius [mm]	240	55.3026	6.47	0.1535	1.66118	4.51
Dist. Focus-Diverg. Slit [mm]	100	58.1472	2.27	0.307	1.58651	1.58
Incident Beam Monochromator	No	59.3517	3.15	0.307	1.55716	2.2
Spinning	Yes	60.3072	5.31	0.2047	1.53475	3.71
		61.3543	11.23	0.2558	1.51105	7.83
		63.7174	6.53	0.3582	1.46059	4.55
		64.7449	9.95	0.307	1.43987	6.94
		67.9118	10.07	0.2047	1.38024	7.03
		72.708	4.56	0.307	1.30056	3.18

**hetearolite 38**		Pos. [°2θ]	Height [cts]	FWHM Left [°2θ]	d-spacing [Å]	Rel. Int. [%]
Scan Axis	Gonio	29.505	19.77	0.1535	3.0275	43.5
Start Position [°2θ]	10.0066	33.2123	25.45	0.2558	2.69755	55.99
End Position [°2θ]	79.9856	36.5357	45.46	0.1279	2.45945	100
Step Size [°2θ]	0.013	59.1671	11.99	0.307	1.56157	26.38
Scan Step Time [s]	37.995	61.0365	13.47	0.307	1.51815	29.64
Scan Type	Continuous					
PSD Mode	Scanning					
PSD Length [°2θ]	2					
Offset [°2θ]	0					
Divergence Slit Type	Fixed					
Divergence Slit Size [°]	0.0573					
Specimen Length [mm]	10					
Measurement Temperature [°C]	25					
Anode Material	Cu					
K-Alpha1 [Å]	1.5406					
K-Alpha2 [Å]	1.54443					
K-Beta [Å]	1.39225					
K-A2 / K-A1 Ratio	0.5					
Generator Settings	40 mA, 45 kV					
Diffractometer Type	11171041					
Diffractometer Number	0					
Goniometer Radius [mm]	240					
Dist. Focus-Diverg. Slit [mm]	100					
Incident Beam Monochromator	No					
Spinning	Yes					

**hydrozincite 41**		Pos. [°2θ]	Height [cts]	FWHM Left [°2θ]	d-spacing [Å]	Rel. Int. [%]
Scan Axis	Gonio	13.335	67.3	0.0768	6.63968	100
Start Position [°2θ]	10.0091	22.403	4.04	0.4093	3.96843	6
End Position [°2θ]	79.9881	24.479	11.58	0.1535	3.63642	17.2
Step Size [°2θ]	0.013	27.269	0.69	0.4605	3.27035	1.02
Scan Step Time [s]	8.67	28.549	21.79	0.2047	3.1266	32.38
Scan Type	Continuous	30.013	3.14	0.307	2.97735	4.66
PSD Mode	Scanning	30.872	3.15	0.8187	2.89644	4.68
PSD Length [°2θ]	2	31.515	6.06	0.2047	2.83885	9
Offset [°2θ]	0	33.156	23.06	0.2047	2.70199	34.26
Divergence Slit Type	Fixed	33.522	11.35	0.1535	2.67327	16.86
Divergence Slit Size [°]	0.0573	34.871	5.85	0.4093	2.57295	8.69
Specimen Length [mm]	10	36.408	17.09	0.2558	2.46774	25.39
Measurement Temperature	25	37.611	4.04	0.1279	2.39151	6
Anode Material	Cu	39.325	4.39	0.4093	2.29118	6.53
K-Alpha1 [Å]	1.5406	41.194	2.35	0.8187	2.19141	3.49
K-Alpha2 [Å]	1.54443	43.755	1.18	0.614	2.06894	1.75
K-Beta [Å]	1.39225	45.159	3.1	0.307	2.00783	4.6
K-A2 / K-A1 Ratio	0.5	47.749	2.59	0.614	1.90479	3.84
Generator Settings	40 mA, 45	54.409	2.44	0.4093	1.68632	3.62
Diffractometer Type	11171041	58.789	4.84	0.307	1.57071	7.19
Diffractometer Number	0	63.520	5.03	0.307	1.46464	7.48
Goniometer Radius [mm]	240					
Dist. Focus-Diverg. Slit	100					
Incident Beam	No					
Spinning	Yes					

**junitiote 31**		Pos. [°2θ]	Height [cts]	FWHM Left [°2θ]	d-spacing [Å]	Rel. Int. [%]
Scan Axis	Gonio	11.3684	64.87	0.0895	7.78368	28.15
Start Position [°2θ]	10.0066	14.1667	230.4	0.0895	6.25185	100
End Position [°2θ]	79.9856	18.8769	49.17	0.0768	4.70119	21.34
Step Size [°2θ]	0.013	19.5395	46.16	0.1023	4.54323	20.04
Scan Step Time [s]	37.995	20.7821	98.61	0.0384	4.27431	42.8
Scan Type	Continuous	22.4378	30.45	0.1279	3.9625	13.22
PSD Mode	Scanning	22.9025	9.47	0.1535	3.88315	4.11
PSD Length [°2θ]	2	24.9011	19.96	0.1535	3.57583	8.67
Offset [°2θ]	0	25.2295	112.86	0.0384	3.53002	48.98
Divergence Slit Type	Fixed	26.5674	11.6	0.0768	3.35521	5.03
Divergence Slit Size [°]	0.0573	27.6625	62.28	0.0512	3.22483	27.03
Specimen Length [mm]	10	28.5247	67.1	0.0384	3.12928	29.12
Measurement Temperature [°C]	25	29.9691	137.11	0.0384	2.98168	59.51
Anode Material	Cu	30.4246	7.36	0.2047	2.93806	3.19
K-Alpha1 [Å]	1.5406	31.7614	146.97	0.0936	2.81506	63.79
K-Alpha2 [Å]	1.54443	31.8318	91.11	0.0468	2.81598	39.55
K-Beta [Å]	1.39225	35.3356	59.9	0.1248	2.53807	26
K-A2 / K-A1 Ratio	0.5	35.544	151.47	0.0468	2.52366	65.74
Generator Settings	40 mA, 45	35.6333	76.87	0.0624	2.5238	33.36
Diffractometer Type	11171041	36.0177	17.72	0.3744	2.49156	7.69
Diffractometer Number	0	38.2357	57.95	0.0468	2.35197	25.15
Goniometer Radius [mm]	240	40.1623	41.25	0.0468	2.24347	17.9
Dist. Focus-Diverg. Slit [mm]	100	42.8728	7.93	0.312	2.10771	3.44
Incident Beam Monochromator	No	44.8277	5.57	0.312	2.02022	2.42
Spinning	Yes	46.0204	19.55	0.0936	1.9706	8.49
		48.5569	24.86	0.0468	1.87342	10.79
		49.7147	2.56	0.3744	1.83247	1.11
		52.5658	18.64	0.0936	1.7396	8.09
		54.7589	2.44	0.312	1.67499	1.06
		57.1845	4.75	0.312	1.60957	2.06
		58.0768	42.75	0.0468	1.58695	18.56
		58.4782	47.21	0.0468	1.57701	20.49
		59.0259	48.62	0.0624	1.56368	21.1
		60.0113	30.96	0.078	1.54033	13.44
		60.1887	18.41	0.0936	1.54004	7.99
		62.231	4.53	0.4992	1.49062	1.97
		63.2321	4.83	0.4992	1.46941	2.09
		64.7041	2.04	0.3744	1.43949	0.88
		70.7082	13.37	0.1248	1.33125	5.8
		72.8633	1.71	0.3744	1.2971	0.74

**legrandite 29**		Pos. [°2θ]	Height [cts]	FWHM Left [°2θ]	d-spacing [Å]	Rel. Int. [%]
Scan Axis	Gonio	7.1949	21.91	0.1023	12.28664	6.66
Start Position [°2θ]	5.0066	13.3732	283.77	0.064	6.621	86.29
End Position [°2θ]	79.9906	15.0649	328.86	0.0512	5.88106	100
Step Size [°2θ]	0.013	17.6589	61.57	0.064	5.0226	18.72
Scan Step Time [s]	37.995	18.2516	15.12	0.1535	4.8608	4.6
Scan Type	Continuous	21.2553	166.31	0.0384	4.18021	50.57
PSD Mode	Scanning	21.6089	30.62	0.1279	4.1126	9.31
PSD Length [°2θ]	2	21.9007	208.35	0.0384	4.05845	63.36
Offset [°2θ]	0	23.6677	28.15	0.0768	3.7593	8.56
Divergence Slit Type	Fixed	24.4722	28.85	0.1535	3.63751	8.77
Divergence Slit Size [°]	0.0573	24.6946	24.69	0.1535	3.60525	7.51
Specimen Length [mm]	10	26.0042	12.36	0.1535	3.42659	3.76
Measurement Temperature [°C]	25	27.0704	57.84	0.0384	3.294	17.59
Anode Material	Cu	28.924	51.17	0.1023	3.08698	15.56
K-Alpha1 [Å]	1.5406	29.7035	33.73	0.2047	3.00773	10.26
K-Alpha2 [Å]	1.54443	30.0992	67.63	0.0384	2.96908	20.57
K-Beta [Å]	1.39225	30.2789	98.07	0.0384	2.95187	29.82
K-A2 / K-A1 Ratio	0.5	30.9203	29.82	0.0768	2.89208	9.07
Generator Settings	40 mA, 45	31.4617	78.66	0.0512	2.84354	23.92
Diffractometer Type	11171041	32.3022	201.86	0.0468	2.76915	61.38
Diffractometer Number	0	32.3841	152.46	0.0384	2.76462	46.36
Goniometer Radius [mm]	240	33.5589	34.02	0.0468	2.66827	10.34
Dist. Focus-Diverg. Slit [mm]	100	34.2029	191.44	0.0468	2.61949	58.21
Incident Beam Monochromator	No	34.2874	119.25	0.0468	2.61972	36.26
Spinning	Yes	34.4012	88.94	0.0468	2.60485	27.05
		34.5263	107.47	0.078	2.59569	32.68
		34.8063	44.83	0.1248	2.57545	13.63
		35.7902	289.18	0.0468	2.50687	87.93
		35.8694	106.99	0.0468	2.50773	32.53
		36.3791	11.54	0.1872	2.46763	3.51
		37.0044	21.99	0.2496	2.42736	6.69
		37.3659	102.89	0.0468	2.40469	31.29
		38.0775	179.79	0.0468	2.36138	54.67
		38.1693	181.73	0.0468	2.35591	55.26
		40.5999	68.91	0.0468	2.2203	20.95
		42.2796	17.76	0.2496	2.13589	5.4
		43.3196	3.69	0.4992	2.087	1.12
		46.1625	7.74	0.3744	1.96487	2.35
		48.6557	10.91	0.3744	1.86985	3.32
		49.2046	9.75	0.1872	1.85027	2.96
		49.9117	6.45	0.3744	1.8257	1.96
		50.5461	25.83	0.0936	1.80426	7.85
		51.5167	8.12	0.2184	1.77253	2.47
		52.7718	9.83	0.312	1.73329	2.99
		53.9697	29.59	0.0936	1.69761	9

	55.5798	75.28	0.156	1.65218	22.89
	55.7368	118.03	0.0624	1.652	35.89
	56.7373	11.93	0.1872	1.62119	3.63
	57.4655	67.69	0.0468	1.60237	20.58
	58.3467	9.81	0.1872	1.58025	2.98
	59.2348	26.02	0.0936	1.55866	7.91
	59.7573	23.27	0.1872	1.54627	7.08
	60.4342	22.83	0.0936	1.53057	6.94
	62.8637	12.37	0.3744	1.47713	3.76
	65.731	13.64	0.1872	1.41946	4.15
	66.9189	29.09	0.0936	1.39712	8.85
	68.7289	61.24	0.078	1.36468	18.62
	68.9219	68.14	0.078	1.36133	20.72
	71.4351	18.3	0.0936	1.31948	5.56
	72.2944	47.19	0.0624	1.3059	14.35

**Rosasite 35**		Pos. [°2θ]	Height [cts]	FWHM Left [°2θ]	d-spacing [Å]	Rel. Int. [%]
Scan Axis	Gonio	10.0928	1.44	0.1535	8.76437	0.06
Start Position [°2θ]	10.0038	10.5785	1.05	0.8187	8.36301	0.05
End Position [°2θ]	89.9928	13.2617	2305.75	0.064	6.6764	100
Step Size [°2θ]	0.013	16.7394	21.01	0.1023	5.29636	0.91
Scan Step Time [s]	18.87	19.251	13.17	0.1535	4.61066	0.57
Scan Type	Continuous	19.823	54.14	0.064	4.47888	2.35
PSD Mode	Scanning	23.6902	16.99	0.1279	3.75579	0.74
PSD Length [°2θ]	2	24.2677	194.17	0.0512	3.6677	8.42
Offset [°2θ]	0	26.4565	65.72	0.0624	3.36624	2.85
Divergence Slit Type	Fixed	26.5236	48.76	0.0468	3.36622	2.11
Divergence Slit Size [°]	0.0573	27.5649	27.74	0.156	3.23334	1.2
Specimen Length [mm]	10	28.049	25.15	0.156	3.17863	1.09
Measurement Temperature [°C]	25	28.7735	23.22	0.156	3.10023	1.01
Anode Material	Cu	30.0005	16.84	0.1872	2.97616	0.73
K-Alpha1 [Å]	1.5406	31.0649	37.96	0.1872	2.87656	1.65
K-Alpha2 [Å]	1.54443	31.9419	21.25	0.2496	2.79956	0.92
K-Beta [Å]	1.39225	32.9797	36.97	0.1872	2.7138	1.6
K-A2 / K-A1 Ratio	0.5	34.3581	66.04	0.2184	2.60801	2.86
Generator Settings	40 mA, 45	36.1079	61.8	0.0624	2.48554	2.68
Diffractometer Type	11171041	37.1606	17.33	0.2496	2.41751	0.75
Diffractometer Number	0	38.8399	9.94	0.1872	2.31676	0.43
Goniometer Radius [mm]	240	40.0291	26.44	0.0468	2.25063	1.15
Dist. Focus-Diverg. Slit [mm]	100	40.5113	25.49	0.2496	2.22495	1.11
Incident Beam Monochromator	No	42.1185	8.37	0.3744	2.14369	0.36
Spinning	Yes	43.0732	2.97	0.8736	2.09836	0.13
		47.2303	11.84	0.2496	1.92291	0.51
		48.2588	5.05	0.2496	1.8843	0.22
		49.2508	10.5	0.2496	1.84864	0.46
		49.9776	16.52	0.3744	1.82345	0.72
		52.289	4.99	0.624	1.74815	0.22
		53.5205	8.94	0.1872	1.71079	0.39
		54.2077	109.35	0.078	1.69072	4.74
		54.3562	51.47	0.0936	1.69064	2.23
		55.4977	21.15	0.4992	1.65443	0.92
		57.7102	12.02	0.3744	1.59616	0.52
		59.8373	9.72	0.3744	1.5444	0.42
		61.5008	10.76	0.4992	1.50655	0.47
		65.478	3.89	0.7488	1.42433	0.17
		67.9478	11.63	0.2496	1.37845	0.5
		72.2305	2.24	0.7488	1.3069	0.1
		76.1577	1.82	0.7488	1.24897	0.08
		77.5285	2.13	0.4992	1.23028	0.09
		79.1542	4.22	0.3744	1.20904	0.18
		82.6666	1.91	0.9984	1.16635	0.08
		86.0777	2.3	0.7488	1.12865	0.1

**scholizite 5**		Pos. [°2θ]	Height [cts]	FWHM Left [°2θ]	d-spacing [Å]	Rel. Int. [%]
Scan Axis	Gonio	10.5177	633.87	0.064	8.41125	100
Start Position [°2θ]	10.0066	13.217	7.31	0.0768	6.69888	1.15
End Position [°2θ]	79.9856	19.8038	20.72	0.0768	4.48319	3.27
Step Size [°2θ]	0.013	20.8921	86.41	0.1023	4.25206	13.63
Scan Step Time [s]	8.67	24.0312	4.25	0.4093	3.70326	0.67
Scan Type	Continuous	26.3668	15.99	0.1279	3.38028	2.52
PSD Mode	Scanning	27.6118	4.91	0.307	3.23063	0.77
PSD Length [°2θ]	2	28.228	19.22	0.1023	3.1615	3.03
Offset [°2θ]	0	28.8339	14.78	0.1023	3.09643	2.33
Divergence Slit Type	Fixed	31.3851	27.72	0.1535	2.85031	4.37
Divergence Slit Size [°]	0.0573	32.083	126.14	0.0384	2.78988	19.9
Specimen Length [mm]	10	33.5402	13.52	0.2047	2.67193	2.13
Measurement Temperature [°C]	25	36.4177	8.3	0.2047	2.46714	1.31
Anode Material	Cu	37.9473	4.33	0.1535	2.37114	0.68
K-Alpha1 [Å]	1.5406	38.8781	7.55	0.1535	2.31649	1.19
K-Alpha2 [Å]	1.54443	39.9689	28.41	0.0468	2.25389	4.48
K-Beta [Å]	1.39225	42.289	3.22	0.1535	2.13721	0.51
K-A2 / K-A1 Ratio	0.5	47.8329	5.94	0.2047	1.90165	0.94
Generator Settings	40 mA, 45	50.5953	3.66	0.4093	1.80412	0.58
Diffractometer Type	11171041	53.4756	2.55	0.1535	1.71354	0.4
Diffractometer Number	0	55.4534	4.44	0.1535	1.65702	0.7
Goniometer Radius [mm]	240	60.0449	4.21	0.2047	1.54083	0.66
Dist. Focus-Diverg. Slit [mm]	100	61.5018	2.18	0.307	1.50778	0.34
Incident Beam Monochromator	No	63.4324	2.62	0.307	1.46646	0.41
Spinning	No	78.3155	1.48	0.4093	1.22088	0.23

**smithsonite 26**		Pos. [°2θ]	Height [cts]	FWHM Left [°2θ]	d-spacing [Å]	Rel. Int. [%]
Scan Axis	Gonio	25.148	175.53	0.0895	3.54121	20.24
Start Position [°2θ]	10.0066	32.562	867.42	0.1279	2.74989	100
End Position [°2θ]	79.9856	38.724	99.25	0.0768	2.32535	11.44
Step Size [°2θ]	0.013	42.795	46.5	0.1535	2.1131	5.36
Scan Step Time [s]	18.87	46.601	50.39	0.0768	1.94897	5.81
Scan Type	Continuous	51.363	21.6	0.2558	1.77894	2.49
PSD Mode	Scanning	53.522	175.25	0.1023	1.71215	20.2
PSD Length [°2θ]	2	62.016	44.12	0.1279	1.4965	5.09
Offset [°2θ]	0	65.966	36.18	0.3582	1.41613	4.17
Divergence Slit Type	Fixed	67.886	22.56	0.2558	1.38069	2.6
Divergence Slit Size [°]	0.0573	69.058	6.72	0.4605	1.36009	0.77
Specimen Length [mm]	10	69.788	20.93	0.2047	1.34765	2.41
Measurement Temperature	25	75.394	4.49	0.614	1.26076	0.52
Anode Material	Cu					
K-Alpha1 [Å]	1.5406					
K-Alpha2 [Å]	1.54443					
K-Beta [Å]	1.39225					
K-A2 / K-A1 Ratio	0.5					
Generator Settings	40 mA, 45					
Diffractometer Type	11171041					
Diffractometer Number	0					
Goniometer Radius [mm]	240					
Dist. Focus-Diverg. Slit	100					
Incident Beam	No					
Spinning	No					

**sphalerite 7**		Pos. [°2θ]	Height [cts]	FWHM Left [°2θ]	d-spacing [Å]	Rel. Int. [%]
Scan Axis	Gonio	28.8004	237.45	0.0384	3.09995	19.98
Start Position [°2θ]	10.0066	33.3713	9.79	0.1535	2.68506	0.82
End Position [°2θ]	79.9856	47.7249	1188.15	0.0468	1.90412	100
Step Size [°2θ]	0.013	47.8515	725.18	0.0624	1.89938	61.03
Scan Step Time [s]	8.67	56.5655	87.73	0.0624	1.62571	7.38
Scan Type	Continuous	59.4158	2.72	0.307	1.55563	0.23
PSD Mode	Scanning	76.9094	21.67	0.1248	1.23863	1.82
PSD Length [°2θ]	2					
Offset [°2θ]	0					
Divergence Slit Type	Fixed					
Divergence Slit Size [°]	0.0573					
Specimen Length [mm]	10					
Measurement Temperature [°C]	25					
Anode Material	Cu					
K-Alpha1 [Å]	1.5406					
K-Alpha2 [Å]	1.54443					
K-Beta [Å]	1.39225					
K-A2 / K-A1 Ratio	0.5					
Generator Settings	40 mA, 45					
Diffractometer Type	11171041					
Diffractometer Number	0					
Goniometer Radius [mm]	240					
Dist. Focus-Diverg. Slit [mm]	100					
Incident Beam Monochromator	No					
Spinning	No					

**tarbuttite 8**		Pos. [°2θ]	Height [cts]	FWHM Left [°2θ]	d-spacing [Å]	Rel. Int. [%]
Scan Axis	Gonio	10.3879	1.36	0.4093	8.51605	0.26
Start Position [°2θ]	10.0066	14.5882	521.87	0.2047	6.07218	100
End Position [°2θ]	79.9856	16.6113	10.14	0.307	5.3369	1.94
Step Size [°2θ]	0.013	19.5588	3.05	0.614	4.5388	0.59
Scan Step Time [s]	8.67	24.3423	27.17	0.307	3.65662	5.21
Scan Type	Continuous	27.4784	11.11	0.307	3.24602	2.13
PSD Mode	Scanning	29.1847	41	0.307	3.06	7.86
PSD Length [°2θ]	2	30.2596	29.95	0.2047	2.95371	5.74
Offset [°2θ]	0	31.3155	38.06	0.307	2.85648	7.29
Divergence Slit Type	Fixed	32.4231	150.55	0.2047	2.76139	28.85
Divergence Slit Size [°]	0.0573	33.2931	5.52	0.2047	2.69119	1.06
Specimen Length [mm]	10	35.6929	10.55	0.2047	2.51556	2.02
Measurement Temperature [°C]	25	36.4703	8.26	0.307	2.46371	1.58
Anode Material	Cu	37.4051	9.83	0.2047	2.40426	1.88
K-Alpha1 [Å]	1.5406	38.4598	24.52	0.307	2.34071	4.7
K-Alpha2 [Å]	1.54443	39.7348	1.11	0.2047	2.2685	0.21
K-Beta [Å]	1.39225	40.8034	2.53	0.2047	2.21152	0.49
K-A2 / K-A1 Ratio	0.5	43.2779	2.15	0.2047	2.09064	0.41
Generator Settings	40 mA, 45	44.2757	184.06	0.2047	2.04582	35.27
Diffractometer Type	11171041	45.1203	5.42	0.2047	2.00946	1.04
Diffractometer Number	0	46.9296	4.17	0.2047	1.93612	0.8
Goniometer Radius [mm]	240	49.4212	4.63	0.2047	1.84419	0.89
Dist. Focus-Diverg. Slit [mm]	100	50.3484	1.41	0.2047	1.81238	0.27
Incident Beam Monochromator	No	50.9868	2.79	0.2047	1.79118	0.54
Spinning	No	52.6481	3.31	0.2558	1.73851	0.63
		53.9399	2	0.2558	1.69988	0.38
		55.2293	0.39	0.2047	1.66321	0.08
		56.8164	3.11	0.2047	1.62047	0.6
		58.1316	1.34	0.2047	1.5869	0.26
		58.7635	12.69	0.2047	1.57133	2.43
		59.4201	3.24	0.2047	1.55553	0.62
		60.1424	27.78	0.2047	1.53856	5.32
		61.7527	6.59	0.2047	1.50225	1.26
		62.4692	9.43	0.2047	1.48673	1.81
		63.2452	0.89	0.2047	1.47035	0.17
		64.1225	1	0.5117	1.45234	0.19
		64.8176	1.01	0.2047	1.43843	0.19
		67.6487	3.4	0.307	1.38496	0.65
		70.8833	0.37	0.4093	1.32949	0.07
		72.6084	2.55	0.307	1.3021	0.49
		75.4744	3.19	0.5117	1.25962	0.61
		77.6056	4.68	0.307	1.23027	0.9

**willemite 9**		Pos. [°2θ]	Height [cts]	FWHM Left [°2θ]	d-spacing [Å]	Rel. Int. [%]
Scan Axis	Gonio					
Start Position [°2θ]	10.0066	12.7399	45.2	0.0768	6.94863	36.18
End Position [°2θ]	79.9856	22.1195	51.81	0.0895	4.0188	41.47
Step Size [°2θ]	0.013	25.5645	115.31	0.0384	3.48451	92.29
Scan Step Time [s]	18.87	31.3436	120.15	0.0936	2.85162	96.16
Scan Type	Continuous	31.4897	77.98	0.1535	2.84107	62.41
PSD Mode	Scanning	34.0119	124.95	0.0468	2.63376	100
PSD Length [°2θ]	2	38.7536	43.28	0.1279	2.32365	34.64
Offset [°2θ]	0	42.116	3.82	0.307	2.14559	3.05
Divergence Slit Type	Fixed	44.9932	8.71	0.2047	2.01484	6.97
Divergence Slit Size [°]	0.0573	46.9297	9.43	0.307	1.93612	7.55
Specimen Length [mm]	10	48.8331	50.29	0.0768	1.86501	40.25
Measurement Temperature [°C]	25	54.1686	5.97	0.307	1.69325	4.77
Anode Material	Cu	55.8512	7.39	0.4093	1.64616	5.92
K-Alpha1 [Å]	1.5406	57.5472	13.16	0.307	1.60161	10.53
K-Alpha2 [Å]	1.54443	59.2659	6.8	0.4093	1.55921	5.44
K-Beta [Å]	1.39225	60.8715	5.34	0.4605	1.52187	4.28
K-A2 / K-A1 Ratio	0.5	65.493	78.06	0.0936	1.42404	62.48
Generator Settings	40 mA, 45	65.6881	43.15	0.0936	1.42382	34.54
Diffractometer Type	11171041	68.5112	33.88	0.156	1.36848	27.11
Diffractometer Number	0	70.1529	14.13	0.4992	1.34042	11.31
Goniometer Radius [mm]	240					
Dist. Focus-Diverg. Slit [mm]	100					
Incident Beam Monochromator	No					
Spinning	No					

**willemite 25**		Pos. [°2θ]	Height [cts]	FWHM Left [°2θ]	d-spacing [Å]	Rel. Int. [%]
Scan Axis	Gonio					
Start Position [°2θ]	10.0066	21.9177	20.14	0.1279	4.05534	5.27
End Position [°2θ]	79.9856	22.2964	81.64	0.0895	3.98731	21.37
Step Size [°2θ]	0.013	25.7989	382.04	0.0468	3.45052	100
Scan Step Time [s]	8.67	25.8709	237.59	0.0468	3.44964	62.19
Scan Type	Continuous	31.7111	93.99	0.078	2.81941	24.6
PSD Mode	Scanning	34.2462	160.56	0.0624	2.61628	42.03
PSD Length [°2θ]	2	38.9991	70.29	0.078	2.30767	18.4
Offset [°2θ]	0	40.9551	6.41	0.3744	2.20186	1.68
Divergence Slit Type	Fixed	42.3875	8.53	0.2496	2.1307	2.23
Divergence Slit Size [°]	0.0573	45.2936	17.27	0.2496	2.00052	4.52
Specimen Length [mm]	10	47.1959	51.03	0.0936	1.92423	13.36
Measurement Temperature [°C]	25	49.0919	71.53	0.0936	1.85425	18.72
Anode Material	Cu	54.4743	6.95	0.312	1.68307	1.82
K-Alpha1 [Å]	1.5406	56.1265	10.31	0.3744	1.63738	2.7
K-Alpha2 [Å]	1.54443	57.7679	34.37	0.1248	1.5947	9
K-Beta [Å]	1.39225	60.9935	53.83	0.1248	1.51786	14.09
K-A2 / K-A1 Ratio	0.5	65.793	64.03	0.1248	1.41828	16.76
Generator Settings	40 mA, 45	67.3374	7.5	0.3744	1.38945	1.96
Diffractometer Type	11171041	68.8288	9.73	0.4992	1.36294	2.55
Diffractometer Number	0	70.489	25.09	0.5616	1.33485	6.57
Goniometer Radius [mm]	240	76.2204	5.62	0.7488	1.2481	1.47
Dist. Focus-Diverg. Slit [mm]	100	79.0321	15.18	0.1872	1.2106	3.97
Incident Beam Monochromator	No					
Spinning	No					
